# Transcriptomic analysis of the phytopathogenic oomycete *Phytophthora cactorum* provides insights into infection-related effectors

**DOI:** 10.1186/1471-2164-15-980

**Published:** 2014-11-18

**Authors:** Xiao-Ren Chen, Bo-Yue Zhang, Yu-Ping Xing, Qi-Yuan Li, Yan-Peng Li, Yun-Hui Tong, Jing-You Xu

**Affiliations:** College of Horticulture and Plant Protection, Yangzhou University, Yangzhou, 225009 China

**Keywords:** *Phytophthora cactorum*, Transcriptome, TribeMCL, Effector, WY-domain, Transcriptional profile, Transient expression, Plant cell death, Polymorphism

## Abstract

**Background:**

*Phytophthora cactorum*, a hemibiotrophic oomycete pathogen, can cause destructive diseases on numerous crops worldwide, leading to essential economic losses every year. However, little has been known about its molecular pathogenicity mechanisms. To gain insight into its repertoire of effectors, the *P. cactorum* transcriptome was investigated using Illumina RNA-seq.

**Results:**

We first demonstrated an *in vitro* inoculation method that can be used to mimic natural cyst germination on host plants. Over 28 million cDNA reads were obtained for five life cycle stages (mycelium, sporangium, zoospore, cyst and germinating cyst) and *de novo* assembled into 21,662 unique genes. By comparisons with 11 public databases, 88.99% of the unique genes were annotated, including 15,845 mapped to the gene models of the annotated relative *Phytophthora infestans*. Using TribeMCL, 5,538 gene families conserved across *P. cactorum* and other three completely sequenced *Phytophthora* pathogen species were determined. *In silico* analyses revealed that 620 *P. cactorum* effector homologues including 94 RXLR effector candidates matched known or putative virulence genes in other oomycetes. About half of the RXLR effector candidates were predicted to share a conserved structure unit, termed the WY-domain fold. A subset of the effector genes were checked and validated by PCR amplification. Transcriptional experiments indicated that effector genes were differentially expressed during the life cycle and host infection stages of *P. cactorum*. Ectopic expression in *Nicotiana benthamiana* revealed that RXLR, elicitin and NLP effectors can trigger plant cell death. These effectors are highly conserved across oomycete species. Single nucleotide polymorphisms for RXLR effectors were detected in a collection of *P. cactorum* isolates from different countries and hosts.

**Conclusions:**

This study demonstrates the comprehensive sequencing, *de novo* assembly, and analyses of the transcriptome of *P. cactorum* life cycle stages. In the absence of genome sequence, transcriptome data is important for infection-related gene discovery in *P. cactorum*, as demonstrated here for the effector genes. The first look at the transcriptome and effector arsenal of *P. cactorum* provides valuable data to elucidate the pathogenicity basis of this broad-host-range pathogen.

**Electronic supplementary material:**

The online version of this article (doi:10.1186/1471-2164-15-980) contains supplementary material, which is available to authorized users.

## Background

The phytopathogenic oomycete *Phytophthora cactorum* (Lebert & Cohn) J. Schröt is capable of infecting an extremely wide range of hosts that span several plant families. It often causes root, collar, and crown rots, as well as foliar and fruit infections. It limits production for many economically important crops worldwide such as strawberry, apple, pear and rhododendron [1, 2]. Additionally, *P. cactorum* is homothallic and produces sexual oospores that can survive for many years in the soil, which makes it more difficult to control the pathogen [1]. At present, there is no effective chemical or cultural strategy available to limit diseases when environmental conditions become warm and wet.

As with many *Phytophthora* pathogens, this species is especially problematic in low-lying or wet field conditions because infection often occurs through the release from sporangia of motile, flagellate and wall-less zoospores that can swim chemotactically or electrotactically toward host plants [1, 3]. Once docking at the potential infection sites, zoospores encyst after shedding their flagella and bind to plant surfaces by means of adhesive glycoproteins [3]. Cysts germinate through extending a germ tube to penetrate the plant surface by enzymatic and mechanical means [3]. Subsequently, host colonization occurs through the growth of a coenocytic mycelium [3]. The various steps of the life cycle of *Phytophthora* have been studied at the cytological level [3]. Also molecular studies have demonstrated that these different stages require specific expression of many genes [4–9]. These life cycle stages of the pathogens are likely to be rich in molecules involved in establishment of infection and elicitation of plant defenses. Hence, the unraveling of the molecular processes regulating the life cycle stages of *Phytophthora* is important to identify determinants of pathogenesis and improve control strategies.

Recent molecular studies of *Phytophthora* pathogens have focused on secreted effectors because of increasing evidence that these diverse proteins manipulate the defense responses of host plants [10, 11]. The genomes of several *Phytophthora* species have been sequenced, including *P. sojae*, *P. ramorum*, *P. infestans* and *P. capsici*[12–14]. The sequencing and analyses of the genomes of other *Phytophthora* pathogens such as *P. parasitica*, *P. cinnamomi* are currently being performed (Broad Institute and JGI, respectively). Transcriptome studies using different approaches such as RNA-seq and microarrays have been conducted to understand the pathogens and/or the interaction with their hosts [9, 15–17]. All of this represents a rich trove of information on the effector repertoires of these pathogens. Various types of secreted effectors are predicted to act in the apoplast (apoplastic effectors) or cytoplasm (cytoplasmic effectors) [10, 18]. Apoplastic effectors, including elicitins, NLPs (Nep1-like proteins), PcF-like proteins, CBEL (Cellulose Binding, Elicitor, and Lectin-like) proteins and enzyme inhibitors, are located at the interface between pathogens and hosts and execute functions outside of the host cell [10]. Elicitins, one type of pathogen-associated molecular patterns (PAMPs), can trigger plant cell death (PCD) response, normally known as hypersensitive reaction (HR). These extracellular proteins share a 98-amino-acid domain with a core of six conserved cysteines in the C-terminal domain [10]. NLPs can cause PCD in dicotyledonous plants and are identified by the presence of a common NPP1 domain [19, 20]. The PcF-like toxin family represents one group of small cysteine-rich proteins from *Phytophthora* species and is thought to have a toxic effect on plants [10, 21]. It has been originally named after the protein effector PcF (*P. cactorum*-*Fragaria*), a 52 amino acid phytotoxic necrosis-inducing protein [22].

Cytoplasmic effectors are able to translocate inside host cells where they interfere with the host physiological functions including defense responses [10]. One family of cell-entering effector proteins (called RXLR effectors) shares an N-terminal RXLR amino acid motif (arginine, any residue, leucine, arginine) [23, 24]. This motif is believed to assist in the translocation of the proteins into the host’s cytoplasm where the effectors suppress basal host resistance or activate effector-triggered immunity (ETI) depending on the host genotype [10, 23, 24]. Host translocation may also occur with variations of the RXLR motif (such as QXLR, GXLR) [25–29]. However, how such effectors cross several biological interfaces to reach the host cytoplasm remains an unclear and debated area of oomycete research [30]. Nevertheless, the importance of these proteins is underlined by the finding that all oomycete Avirulence genes identified to date encode RXLR effectors [18, 31–34]. Due to selection pressure from the hosts, the Avirulence effectors show extensive variations, including amino acid changes indicative of strong positive selection, gene truncations and deletions, and transcriptional silencing [35, 36]. The effector activity resides in the C-terminal regions of RXLR effectors (the “effector domain”) that are typically under positive selection [35, 37]. Despite the extensive sequence diversity, the C-terminal regions of about half of RXLR effectors display a conserved core α-helical fold (termed the “WY-domain”) that probably tolerates considerable plasticity [38, 39]. In addition to the RXLR effectors, other cytoplasmically-localized effectors have been identified in *Phytophthora* species [10, 18]. The Crinkler (CRN for CRinkling and Necrosis) protein family produces a leaf crinkling and necrosis phenotype when expressed in *Nicotiana benthamiana*[40]. Recent studies revealed that some CRN effectors target host nuclei [41, 42]. A possible role for some CRNs towards *Phytophthora* virulence has been suggested [43, 44]. Intriguingly, a substantial number of phosphorylated CRNs were recently found in six life stages of *P. infestans*, from hyphae to appressoria, implying that some members of CRN family could have functions other than to serve as effectors during infection [45]. Although the effector repertoire is generally highly divergent between species, features such as motifs or domains [10, 35, 38, 39] shared by effectors in different *Phytophthora* species allow rapid identification of effector candidates from genome sequences.

Despite its economic importance, molecular studies of *P. cactorum* have significantly lagged behind studies on other *Phytophthora* species. Studies of the molecular basis of the pathogenicity of *P. cactorum* are limited to the chemical identification of α-elicitin [46] and the phytotoxic protein PcF [21, 22, 47, 48]. Changes in *P. cactorum* gene expression profiles prior to and during strawberry infection were recently studied using two approaches (suppression subtractive hybridization and differential display) [8]. A set of differentially expressed genes including 11 RXLR candidate genes was identified, but no further verification or functional characterization were conducted. Recent advancements in sequencing technologies have led to an explosive growth in the analysis of infection-related genes of oomycete plant pathogens [9, 15, 49–52]. The application of next generation sequencing techniques in non-model species promises to collect large amount of data necessary for functional studies rapidly and cost-effectively.

In order to gain more insights into the molecular mechanisms of *P. cactorum* pathogenicity, in the present study we report on the Illumina sequencing, *de novo* assembly, annotation and analysis of the *P. cactorum* transcriptome in important life cycle stages. A large number of genes and gene families were identified using different methods. We investigated the potential effector arsenal including RXLR effectors. The RXLR candidate effector dataset was searched for the conserved structure unit, WY-domain fold. Transcript levels of potential effector genes were monitored during the developmental and host infection stages of *P. cactorum*. Heterologous expression revealed that some effectors could elicit PCD. Sequence polymorphisms and positively selected sites in RXLR effector candidates were also analyzed. In total, the work described herein provides a crucial foundation for further dissection of genes relevant to virulence in this broad-host-range phytopathogen.

## Results

### Sampling of *P. cactorum*life cycle stages

**Figure 1:**
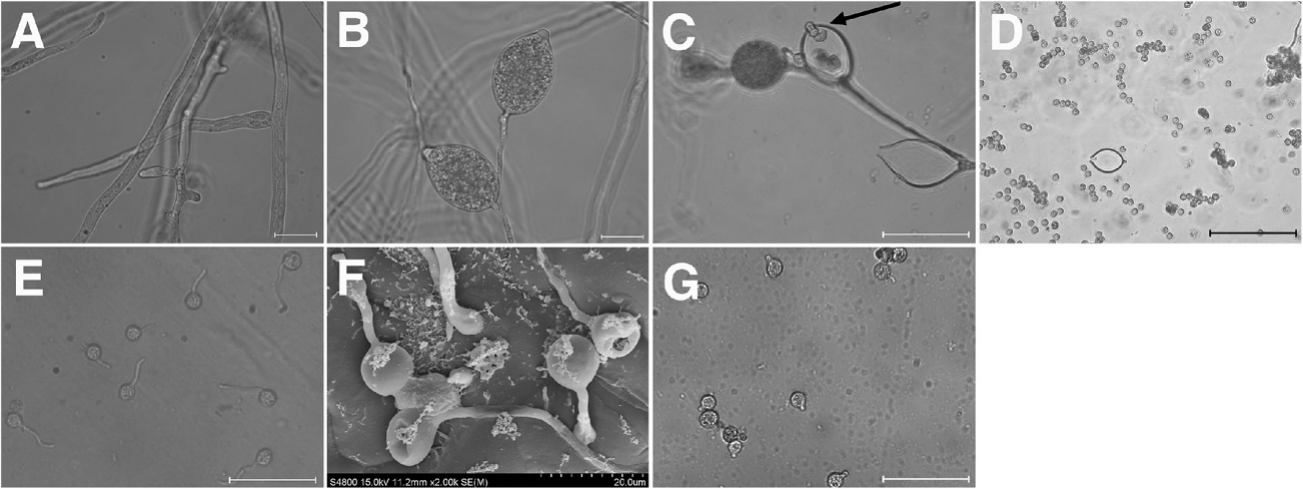
**Life cycle stages of** ***P. cactorum*** **including cysts germinating under different conditions.** Five successive life cycle stages are shown: **(A)** mycelia, **(B)** sporangia, **(C)** released sporangia and zoospores, **(D)** cysts, **(E)**-**(G)** germinating cysts. In panel **(C)**, the arrow indicates a zoospore being released from the sporangium. Cysts were germinated on a cellophane membrane placed on the top of an *N. benthamiana* leaf **(E)**, or directly on *N. benthamiana* leaves **(F)**, or in water **(G)**. In panel **(F)**, the cysts were observed under a Cryoscanning electron microscope (Hitachi S-4800 SEM). The other cell types were observed using an Olympus System Microscope BX53. Photos were taken at 70 **(E)**, 60 **(F)** and 300 **(G)** min post-inoculation. Scale bars: **(A)**, **(B)**, 10 μm; **(C)**, **(E)**, **(G)**, 50 μm; **(D)**, 100 μm.

To gain a global overview of the *P. cactorum* transcriptome and gene activity during the important life stages, we prepared a mixed cDNA sample using RNA from 5 different life stages: mycelia (MY), sporangia (SP), swimming zoospores (ZO), cysts (CY) and germinating cysts with germ tubes (GC) (Figure 1A-E).

**Figure 2:**
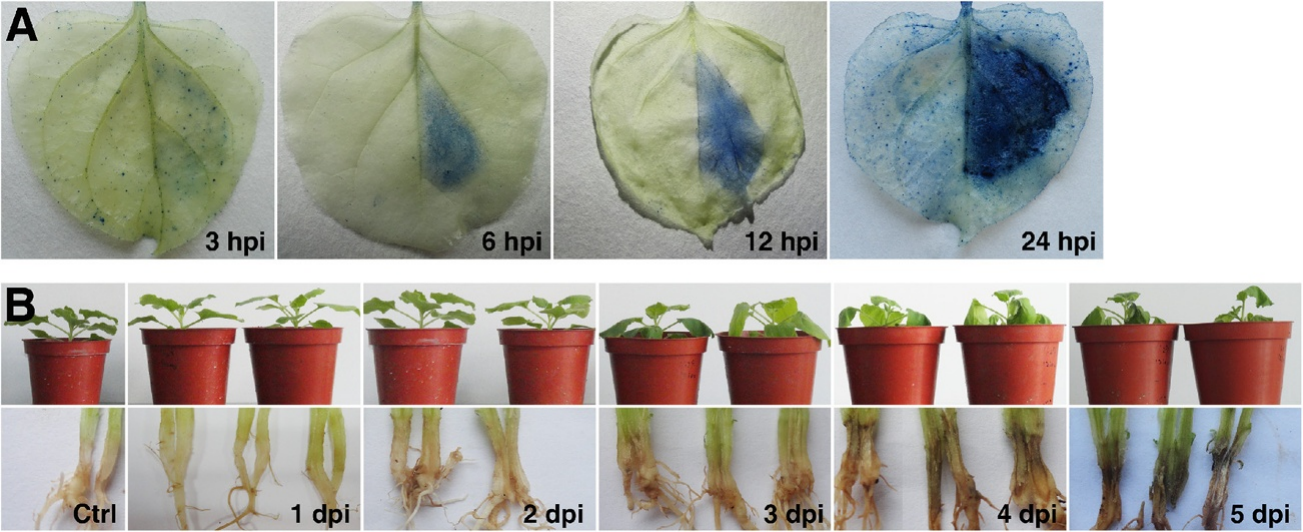
**Disease progressions in** ***N. benthamiana*** **caused by** ***P. cactorum*** **isolate 10300.** Leaves **(A)** or roots **(B)** were inoculated with a *P. cactorum* zoospore suspension. **(A)** Trypan blue-stained leaves at 3, 6, 12 and 24 h post-inoculation (hpi). On each leaf, the left side of the main vein was mock-inoculated with water while the right side was inoculated with zoospores. **(B)** Plants were inoculated by pouring zoospore suspension around the roots. Upper panel, disease development on whole plants 1, 2, 3, 4, 5 days post-inoculation (dpi). Lower panel, root from plants in upper panel experiments were cut open to observe symptoms inside the roots. Ctrl, roots from control plants mock-inoculated with water.

In this study, we found that *P. cactorum* can infect the model plant *N. benthamiana* at least through foliage and roots (Figure 2). The infection by *P. cactorum* reproducibly caused visible leaf cell death, observed at 3, 6, 12, and 24 h post-inoculation (hpi) using trypan blue staining (Figure 2A). In order to investigate early infection events by *P. cactorum* but to avoid the involvement of plant tissue in the pathogen RNA samples for later transcriptome sequencing, a cellophane membrane placed on the *N. benthamiana* leaf was used as a surface for the induction of cyst germination as previously described for *Magnaporthe grisea*[53]. The cysts germinating on both cellophane membranes (Figure 1E) and *N. benthamiana* leaves (Figure 1F) were morphologically similar, in contrast to cysts germinating in water (Figure 1G).

To further test if the cysts germinating under this artificial condition display similar gene expression pattern to that of natural infection, 6 RXLR candidate genes (*PcRXLR6*, *PcRXLR8*, *PcRXLR13*, *PcRXLR16*, *PcRXLR21* and *PcRXLR25,* details described later) were selected for comparison with normalized read counts and real-time quantitative RT-PCR (qRT-PCR). During the transcriptome sequencing period, Illumina RNA-Seq was also utilized to perform massively parallel sequencing of *P. cactorum* MY, ZO and GC in our lab (X-R Chen and B-Y Zhang, unpublished data). About 10 million sequence reads for each library (MY, ZO and GC) were mapped to the assembled reference transcriptome (see below for the transcriptome details), and the transcript level of each gene in different libraries was calculated and normalized to the reads per kilobase of exon model per million mapped reads (RPKM) [54] for comparison. At the same time, expression of these RXLR candidate genes’ mRNA was measured using qRT-PCR under the artificial condition. A parallel qRT-PCR analysis was also performed with the cysts germinating directly on *N. benthamiana* leaves for comparison. In GC versus MY, all these 6 RXLR candidate genes showed the same expression pattern (expressed at a “high, medium or low” level) under artificial condition as during natural infection (Additional file 1). This direct comparison faithfully demonstrated that genes differentially expressed in cysts germinating on cellophane show the same expression pattern as during natural infection, relative to the mycelium.

These results convinced us that cellophane membrane was a valid model for the cyst germination. We did not harvest cysts germinating in water because in the absence of the leaf or membrane surface, cyst germination was significantly slower and non-uniform (Figure 1G). Hence, RNA from the cysts germinating on cellophane membrane was extracted at 60 min post inoculation and mixed equally with the MY, SP, ZO and CY RNA samples for Illumina RNA-Seq.

### Transcriptome sequencing and *de novo*assembly

We used Illumina platform to perform *P. cactorum* transcriptome sequencing. Two biological replicates were generated and sequenced for mixed samples of five life cycle stages. Each sequenced sample yielded 2 × 75 or 2 × 83-bp independent reads from either end of a cDNA fragment (Additional file 2). After filtration of low-quality and adapter sequences, a total of 28,693,892 cleaned cDNA reads (~2.2 Gb) was obtained and combined for analyses hereafter. The percentage of Q20 bases for the clean reads were >94%. An average “G + C” content of above 56% was observed for the *P. cactorum* cDNA sequences (Additional file 2). An overview of the assembled contigs, scaffolds and unigenes is presented in Table 1. Initially, the reads were assembled into 277,262 contigs. After paired-end joining and gap-filling, the contigs were further assembled into 26,324 scaffolds totaling 31,983,660 bases. The final clustering of scaffolds produced 21,662 unique genes (hereafter referred to as unigenes, meaning a scaffold that matches no other scaffolds). More than half of the unigenes (13,071, 60.34%) were longer than 500 bp and 8,257 unigenes (38.12%) were longer than 1 kbp. The distribution of scaffolds and unigenes is shown in Additional file 3. For the randomly fragmented transcriptome, there was a positive relationship between the length of a given unigene and the number of reads assembled into it (Additional file 3). To evaluate the completeness of the transcriptome assembly, we used the Core Eukaryotic Genes Mapping Approach (CEGMA) pipeline [55], with the CEGMA subset of the 248 widely conserved core eukaryotic genes that are likely to have low frequencies of gene family expansion (http://korflab.ucdavis.edu/Datasets/genome_completeness/). Using this tool, 243 (98%) of the 248 CEGMA genes mapped against the *P. cactorum* transcriptome assembly were identified. The high recovery of CEGMA genes suggests a high quality transcriptome assembly [55].

**Table 1:** **Summary of transcriptome assembly for** ***P. cactorum***

Category	Count
Assembled reads	28,693,892
Total base pairs	2,277,839,244
Contigs	277,262
Maximum length of contig (bp)	20,441
Minimum length of contig (bp)	60
Mean length of contigs (bp)	148
Scaffolds	26,324
Total length of scaffolds (bp)	31,983,660
Maximum length of scaffold (bp)	20,441
Minimum length of scaffold (bp)	201
Mean length of scaffolds (bp)	1,215
Scaffold size N50 (bp)	2,007
Unigenes	21,662
Maximum length of unigene (bp)	20,441
Minimum length of unigene (bp)	201
Mean length of unigenes (bp)	1,116
Unigene size N50 (bp)	1,836

### Sequence annotation

The unigenes were annotated by aligning them with the deposited ones in 11 diverse databases including *P. infestans*, *P. sojae*, *Pythium*, NCBI Nr, NCBI Nt, Swiss-Prot, TrEMBL, InterProScan databases (Table 2). The best one was selected from the matches with an *E*-value of less than 10^−5^. In total, 19,277 (88.99%) unigenes could be matched to the genes from at least one of 11 public databases (Table 2). The results demonstrated that of the 21,662 unigenes, 15,845 (73.15%) had significant amino acid sequence matches in the *P. infestans* database and 14,759 (68.13%) retrieved significant hits in the *P. sojae* database while only 7,091 (32.73%) could be mapped to *Pythium* genes. In contrast, 18,624 (85.98%) of unigenes could be matched to the sequences deposited in the NCBI Nr database.

**Table 2:** **Summary of functional annotation of the** ***P. cactorum*** **transcriptome**

Database	Number of annotated unigenes	Percentage (annotated/total number of unigenes)
*P. infestans* Annotation	15845	73.15%
*P. sojae* Annotation	14759	68.13%
*Pythium* Annotation	7091	32.73%
Nr Annotation	18624	85.98%
Nt Annotation	16041	74.05%
InterProScan Annotation	9331	43.08%
TrEMBL Annotation	10512	48.53%
Swissprot Annotation	10859	50.13%
KEGG Annotation	2717	12.54%
GO Annotation	7605	35.11%
COG Annotation	5491	25.35%
Total annotated	19277	88.99%

**Figure 3:**
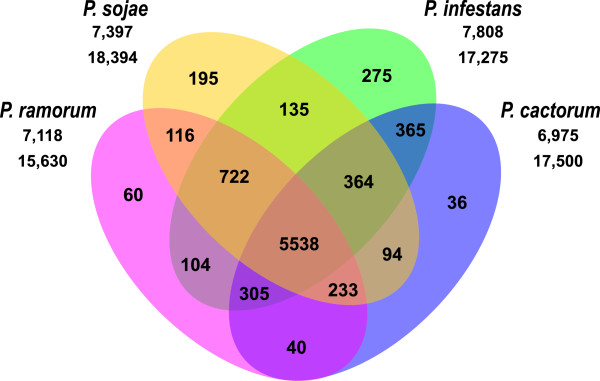
**Four-way-Venn-diagram of the distribution of unique and shared gene families among** ***Phytophthora*** **species.** Homologous proteins in *P. cactorum*, *P. infestans*, *P. sojae* and *P. ramorum* were clustered into gene families using TribeMCL. Numbers in individual sections indicate number of gene families (not genes). Overlapping regions denote groups with at least two proteins of all species that are part of the intersection. The first number under the organism name gives the total number of clusters that the respective organism contributes to; the second number describes the total number of each organism’s gene models/sequences that go into clusters. The difference of the second number to an organism’s total input proteins gives the number of singletons (protein sequences that do not cluster at all in this analysis).

To better define conserved genes in *P. cactorum*, protein sequences from the transcriptome assembly of this species (19,886 input proteins) and three completely sequenced *Phytophthora* pathogens, *P. infestans* (18,138), *P. sojae* (19,027) and *P. ramorum* (15,743), were clustered into gene families based on reciprocal pairwise sequence similarities (Figure 3). In total, 68,799 protein sequences from these four *Phytophthora* species were clustered into 8,582 groups. Of them, 5,538 orthologous groups were shared by all four species. A total of 6,939 orthologous groups for *P. cactorum*, 7,533 for *P. infestans*, 7,202 for *P. sojae*, and 7,058 for *P. ramorum* were generated. About 12% (2,471 out of 19,886) of *P. cactorum* putative proteins did not have orthologues in the other three *Phytophthora* species, of which 85 protein sequences were clustered into 36 paralogous groups within the species (Figure 3)*.* The remaining predicted proteins (2,386) are considered singletons because they do not cluster at all in this analysis.

By 23 March 2014, there had been 774 *P. cactorum* nucleotide sequences including 280 expressed sequence tags (ESTs) deposited in the NCBI Nt database. In order to characterize these sequences, we queried them against our transcriptome assembly. BLASTn comparisons revealed that 421 of 494 (85.2%) nucleotide sequences excluding ESTs were mapped to 21 *P. cactorum* unigenes (Additional file 4). Of 421 mapped sequences, 308 (73.2%) were matched to the same unigene *U10832* (ribosomal RNA gene) (Additional file 4). In contrast, 180 of 280 (64.3%) ESTs were mapped to 157 unigenes (Additional file 5). Out of them, some ESTs may be derived from the same gene and 39 redundant ESTs were mapped to 16 unigenes. Six unknown ESTs turned out to be clustered into ribosomal RNA unigene *U10832* (Additional file 5).

GO annotation in terms of “biological process”, “molecular function” and “cellular component” was conducted for the annotated *P. cactorum* unigenes and, for comparison, 22,658 gene models of *P. infestans*. Based on sequence similarity, 7,605 *P. cactorum* unigenes could be categorized into 47 functional groups. As shown in Additional file 6, the distribution pattern of *P. cactorum* sequences in different GO categories (level 2) was quite similar to that of 9,829 *P. infestans* genes. The unigenes were assigned with one or more GO terms. For “cellular component”, “cell & cell part”, “organelle” and “membrane” terms are dominant. For “molecular function”, genes related to “binding” (6,008, 79.00%) and “catalytic activity” (5,968, 78.47%) are highly represented. Regarding “biological process”, “metabolic process” and “cellular process” are the highly represented GO terms. We found a few genes from terms of “antioxidant activity” (50, 0.65%), “signaling” (580, 7.62%), and “localization” (1,729, 22.74%).

Besides GO analysis, the unigenes were subjected to a search against the COG database for functional prediction and classification. In total, 5,491 of the 18,624 sequences retrieving a hit from the NCBI Nr database could be assigned to COG classifications (Additional file 7). COG-annotated putative proteins were functionally classified into 25 protein families involved in cellular structure, biochemical metabolism, molecular processing, signal transduction and so on (Additional file 7). The cluster for “general function prediction” represents the largest unknown group (1,606, 20.59%), followed by “replication, recombination and repair” (722, 9.26%), “transcription” (606, 7.77%) and “signal transduction mechanisms” (605, 7.76%). The following categories: “extracellular structures” (1, 0.01%), “nuclear structure” (2, 0.03%) and “cell motility” (5, 0.06%), represent the smallest groups. In addition, 219 unigenes were assigned to “defense mechanisms” and 538 unigenes were assigned to “translation, ribosomal structure and biogenesis” (Additional file 7).

To identify the biochemical pathways that are active in *P. cactorum*, we mapped the 18,624 annotated sequences to the reference canonical pathways in KEGG. This is a complementary approach to categorize genes functions with the emphasis on biochemical pathways. The process predicted a total of 275 pathways represented by a total of 2,717 unigenes. A summary of the sequences involved in these pathways is included in Additional file 8. These predicted pathways represented the majority of biochemical pathways for compound biosynthesis, degradation, utilization, and assimilation, and pathways involved in the processes of detoxification and generation of precursor metabolites and energy. The pathways with most representation by unique sequences were “ribosome” (235 members), “chromosome” (224 members) and “spliceosome” (179 members).

### Searching and validation of the *in silico*-predicted *P. cactorum*effector genes

Like other plant oomycete pathogens, *P. cactorum* presumably secretes a battery of virulence proteins to promote infection. The secretome for pathogenicity-related proteins was identified by comparison of *P. cactorum* unigenes against public databases. A total of 620 effector genes with known or putative roles in virulence were identified (Table 3). Notable families of secreted proteins include NLPs, CBELs with carbohydrate binding domains, elicitins and elicitin-like proteins, PcF-like proteins, and protease inhibitors (serine and cysteine) (Table 3). Our data indicate that *P. cactorum* exhibits the same kinds of pathogenicity-related effector proteins that have been found in other oomycetes [10–14].

**Table 3:** **Gene families potentially implicated in plant pathogenesis in** ***P. cactorum***

Gene family	***P. cactorum*** ^a^	***P. infestans*** ^b^	***P. sojae*** ^b^	***P. ramorum*** ^b^	***Pythium ultimum*** ^b^	***Hyaloperonospora arabidopsidis*** ^b^	***Albugo laibachii*** ^b^	***Saprolegnia parasitica*** ^b^
ABC transporters, all	190	156	134	135	140	55	41	129
CBELs	6	4	5	5	3	2	3	40
Crinklers (CRN-family)	64	196	40	8	26	20	3	0
Cutinases	7	4	16	4	0	2	2	0
Cysteine proteases	32	33	67	74	77	33	16	85
Elicitin/Elicitin-like proteins	44	40	57	48	24	15	3	29
Glycosyl hydrolases	122	157	125	114	180	>60	44	74
NPP1-like proteins	31	27	29	40	7	10	0	0
PcF-like proteins	3	16	19	4	3	2	1	1
Pectinesterases	3	11	19	15	0	3	0	0
Protease inhibitors, all	24	38	22	19	15	3	0	7
RXLR effectors	94	563	350	350	0	134	49	0

^a^The number of “real” effector genes in our dataset is yet to be determined though obvious pseudogenes are omitted.

^b^Data from other oomycete species are from Haas et al. [13] for *P. infestans*, Tyler et al. [12] for *P. sojae* and *P. ramorum*, Lévesque et al. [92] for *Pythium ultimum*, Baxter et al. [70] for *H. arabidopsidis*, Kemen et al. [50] for *A. laibachii*, or Jiang et al. [93] for *S. parasitica*. Counts of annotated pseudogenes are omitted.

Recently, many studies have shown a vast repertoire of apoplastic and cytoplasmic effector proteins including RXLR and Crinkler (CRN) families in oomycetes [10–14]. The *P. cactorum* unigene dataset was searched for similarity to known oomycete effectors. This search identified 93 potential RXLR effectors containing an RXLR or RXLR variant motif located downstream of the signal peptide. To find the potential RXLR effectors without similarity to hits in sequenced genomes of oomycete species, all putative ORFs from the assembly were generated and translated to amino acid sequences from both strands. The predicted peptides were analyzed using the RXLR effector identification method [56]. This yielded the same RXLR effector dataset as BLASTx comparison did. Another search for the amino acid motif RXLR was performed on these predicted peptides. HMMER searches employing models described previously [13] yielded five additional potential RXLR effectors. Signal peptide analysis was conducted using SignalP 3.0 and four candidates were excluded because of low SignalP HMM probability (<0.9). These led to the final set of 94 unique potential RXLR effectors (referred to as PcRXLR1 to PcRXLR94; Table 3 & Additional file 9). Phylogenetic analysis of the *Phytophthora* genus has shown that *P. cactorum* lies with *P. infestans* and *P. parasitica* in Clade 1 [57]. Comparison of our RXLR dataset against *P. parasitica* INRA-310 (v2) genome was also performed to double check the effector searching (Additional file 10). We mapped a total of 81 effector candidates to *P. parasitica* RXLR genes, leaving 12 that matched to hypothetical protein-encoding genes and one not mapped to *P. parasitica*. In contrast, 89 mapped to *P. infestans* RXLR genes and only 5 mapped to uncharacterized *P. infestans* genes (3) or no hits (2) (Additional file 10). Four candidates matched to uncharacterized genes or no hit of both genomes at the same time. Those uncharacterized hit proteins were identified as not RXLR effectors because of absence of RXLR motif in the sequences. Among the 94 potential effectors, 46 contained a conserved RXLR motif. Ten contained a variant of RXLR motif (RXLX) that is similar to the Pexel translocation motif of *Plasmodium falciparum* effectors (RXLX^E^/Q_/D_). The details of 64 candidates containing RXLR and/or dEER motif(s) or variants are provided in Table 4. These candidates include 43 intact ORFs and 21 incomplete sequences. The remaining 30 RXLR effector candidates are found not to contain either RXLR or dEER motifs due to short sequences (Additional file 9).

**Table 4:** **Canonical and variant RXLR-dEER motifs and the WY-domain found in** ***P. cactorum*** **transcriptome protein models**

Effector name	Transcriptome unigene ID ^a^	Protein length	SignalP HMM probability ^b^	SignalP NN mean S score ^b^	SignalP length ^b^	RXLR start	RXLR motif ^c^	dEER start	dEER motif ^cd^	Stop codon	Evidence ^e^	WY-domain ^cf^	Seq-from ^f^	Seq-to ^f^	***E*** -value ^f^	Score ^f^
*PcRXLR1*	467	180	0.999	0.950	20	50	RYLR	62	DEER	181	MB	—	—	—	—	—
*PcRXLR2*	3444	276	0.999	0.863	23	51	RSLR	63	NENEER	277	MB	2/2	218	261	4.50e-06	13
*PcRXLR3*	4585	160	0.997	0.907	20	51	RFLR	86	DEER	161	MBPR	1/1	94	141	2.90e-11	29.6
*PcRXLR4*	4911	198	0.999	0.918	18	57	RLLR	71	DDEEER	199	MBR	2/2	138	177	1.70e-06	14.3
*PcRXLR5*	7413	288	0.998	0.955	22	42	RSLR	56	EDNEER	289	MBPR	1/3	106	125	0.00015	8.1
*PcRXLR6*	12534	260	1.000	0.848	20	50	RFLR	68	EER	261	MBPR	—	—	—	—	—
*PcRXLR7*	14177	253	0.998	0.913	16	95	RLLR	114	EER	254	MBPR	—	—	—	—	—
*PcRXLR8*	14674	147	0.995	0.892	25	48	RFLR	66	DDEER	148	MBPR	2/3	89	112	0.00026	7.4
*PcRXLR9*	14719	132	1.000	0.885	23	41	RSLR	50	EER	133	MBPR	1/1	66	114	9.70e-07	15.1
*PcRXLR10*	15159	136	0.998	0.818	23	56	RLLR	71	EEEER	137	MBP	2/2	77	125	4.90e-11	28.8
*PcRXLR11*	15234	171	1.000	0.959	21	54	RFLR	74	EER	172	MBPR	—	—	—	—	—
*PcRXLR12*	15411	333	1.000	0.898	24	29	RGLR	41	EDDE	334	MB	—	—	—	—	—
*PcRXLR13*	15681	407	1.000	0.944	20	52	RFLR	66	EER	408	MBPR	3/4	270	317	4.60e-17	48.1
*PcRXLR14*	16067	358	0.999	0.866	19	49	RFLR	65	EEK	359	MBP	2/3	178	222	1.60e-06	14.4
*PcRXLR15*	17198	503	1.000	0.940	19	52	RFLR	58	DDEER	504	MBP	4/5	355	402	5.20e-20	57.5
*PcRXLR16*	17759	162	1.000	0.877	24	30	RRLR	75	DEQ	163	MBPR	—	—	—	—	—
*PcRXLR17*	17849	349	0.998	0.853	20	42	RSLR	52	EDDEER	350	MBPR	1/4	105	153	2.10e-20	58.7
*PcRXLR18*	17862	244	0.985	0.698	23	55	RFLR	69	EDDEER	245	MB	1/1	180	219	7.80e-11	28.2
*PcRXLR19*	17955	134	1.000	0.967	20	48	RFLR	62	DEER	135	MB	1/1	77	120	7.70e-08	18.6
*PcRXLR20*	18066	171	1.000	0.927	23	58	RLLR	80	EER	172	MBPR	—	—	—	—	—
*PcRXLR21*	18476	142	1.000	0.927	21	50	RSLR	62	DEEEDEEDEER	143	MBPR	—	—	—	—	—
*PcRXLR22*	18992	290	1.000	0.907	21	56	RFLR	62	DDEER	291	MBPR	2/3	155	199	5.30e-08	19.2
*PcRXLR23*	19550	116	0.999	0.887	21	48	RHLR	65	DEER	117	MBPR	—	—	—	—	—
*PcRXLR24*	19979	309	1.000	0.831	24	36	RLLR	45	DEE	310	MB	1/3	69	117	3.70e-18	51.5
*PcRXLR25*	20127	145	0.998	0.798	20	54	RFLR	74	EER	146	MBPR	—	—	—	—	—
*PcRXLR26*	20211	233	0.999	0.926	21	42	RALR	55	EER	234	MB	1/2	90	125	1.70e-10	27.1
*PcRXLR27*	20751	133	0.999	0.862	22	52	RFLR	63	DEER	134	MBPR	—	—	—	—	—
*PcRXLR28*	3199	60	0.999	0.910	24	38	RFLR	—	—	61	MB	—	—	—	—	—
*PcRXLR29*	13894	330	0.983	0.620	25	47	RSLR	—	—	331	MB	—	—	—	—	—
*PcRXLR30*	18832	148	0.975	0.929	25	46	RFLR	—	—	149	MBP	1/1	76	124	4.20e-14	38.6
*PcRXLR31*	20872	211	0.999	0.950	19	73	RKLR	—	—	212	MB	—	—	—	—	—
*PcRXLR32*	1468	120	0.999	0.796	20	43	RYLK	58	DEDR	121	MB	—	—	—	—	—
*PcRXLR33*	1929	143	0.999	0.875	22	46	RFLT	55	DDER	144	MB	1/1	70	118	2.50e-19	55.3
*PcRXLR34*	2443	185	1.000	0.958	20	42	RNLK	58	DEER	186	MB	—	—	—	—	—
*PcRXLR35*	4402	157	1.000	0.948	23	41	RVLL	53	EER	158	MB	2/3	90	121	4.90e-05	9.7
*PcRXLR36*	7520	210	1.000	0.845	25	36	RLLS	52	ER	211	MB	—	—	—	—	—
*PcRXLR37*	14168	143	0.998	0.890	24	43	RKLA	59	EER	144	MB	—	—	—	—	—
*PcRXLR38*	17882	139	0.999	0.845	23	53	RMLK	64	DDEER	140	MB	1/1	86	132	1.40e-07	17.8
*PcRXLR39*	19735	171	0.933	0.768	23	42	RSLK	58	DEDR	172	MB	—	—	—	—	—
*PcRXLR40*	20420	190	0.996	0.924	21	42	RHLK	56	EER	191	MB	—	—	—	—	—
*PcRXLR41*	1130	—	1.000	0.953	20	52	RLLR	65	EER	—	MB	1/2	91	137	5.50e-20	57.4
*PcRXLR42*	6874	—	1.000	0.926	20	40	RSLR	52	DDEEER	—	MB	—	—	—	—	—
*PcRXLR43*	11787	—	1.000	0.817	23	42	RHLR	55	EE	—	MB	—	—	—	—	—
*PcRXLR44*	13785	—	1.000	0.882	22	46	RFLR	55	DEER	—	MB	—	—	—	—	—
*PcRXLR45*	16936	—	0.995	0.856	24	35	RMLR	46	EEE	—	MB	—	—	—	—	—
*PcRXLR46*	3185	—	—	—	—	15	RLLW	36	EDDEEER	—	MB	1/2	57	104	2.60e-08	20.1
*PcRXLR47*	3331	—	—	—	—	16	RSLR	31	EER	—	MB	—	—	—	—	—
*PcRXLR48*	6076	—	—	—	—	53	RLLR	63	EE	—	MB	—	—	—	—	—
*PcRXLR49*	8191	—	—	—	—	20	RYLR	30	EDEDR	—	MB	—	—	—	—	—
*PcRXLR50*	8328	—	—	—	—	36	RRLR	52	EER	—	MB	1/1	66	114	8.30e-15	40.9
*PcRXLR51*	8440	—	—	—	—	21	RYLR	39	DEER	—	MB	1/1	101	142	1.20e-06	14.8
*PcRXLR52*	11059	—	—	—	—	18	RLLR	28	EN	—	MB	—	—	—	—	—
*PcRXLR53*	11691	—	—	—	—	29	RSLR	39	DEER	—	MB	1/1	46	74	6.00e-08	19
*PcRXLR54*	12599	—	—	—	—	28	RYLR	44	EER	—	MB	—	—	—	—	—
*PcRXLR55*	1675	—	—	—	—	—	—	3	EER	—	MB	—	—	—	—	—
*PcRXLR56*	6278	—	—	—	—	—	—	11	EER	—	MB	1/1	35	66	9.50e-06	12
*PcRXLR57*	10850	—	—	—	—	—	—	1	DDE	—	MB	2/3	120	167	4.40e-16	44.9
*PcRXLR58*	1999	—	—	—	—	—	—	3	DEER	265	MB	3/4	141	189	1.90e-13	36.5
*PcRXLR59*	18301	—	—	—	—	—	—	18	DDDEER	440	MB	4/5	305	353	2.50e-11	29.8
*PcRXLR60*	20091	—	—	—	—	—	—	1	EDEER	314	MB	—	—	—	—	—
*PcRXLR61*	21078	—	—	—	—	18	RGLR	28	ND	307	MB	—	—	—	—	—
*PcRXLR62*	17023	160	0.986	0.409	25	54	RFLR	65	DNEER	161	MB	—	—	—	—	—
*PcRXLR63*	17457	134	0.999	0.925	19	37	GILR	51	EEER	135	MB	—	—	—	—	—
*PcRXLR64*	13871	117	0.996	0.772	21	45	KLLR	61	EQEER	118	MB	—	—	—	—	—

^a^Effectors were predicted from the unigene sequences.

^b^Hidden Markov model (HMM) probability, NN mean S score, and signal peptide length were predicted using SignalP v3.0.

^c^All sequence coordinates are given in amino acid residues.

^d^Determined as two or more D,E,N and/or Q residues followed by a R or K residue: [DENQ]{2,}[RK].

^e^Evidence codes for RXLR gene prediction: M (motif search), B (BLAST comparison), P (PCR validation), and R (RT-PCR).

^f^WY-domain was predicted using the HMM for the WY-domain as described in [38] with the HMM score cut-off of 0.0. The “—” means that no WY-domain was predicted.

We performed similar searches to identify CRN effectors in *P. cactorum*. In total, 64 potential CRN proteins were identified (hereafter PcCRN1 to PcCRN64; Table 3 and Additional file 11). Three other PcCRNs were not picked up due to too short ORF (<300 nt) [58] or lacked the characteristic N-terminal ~50-amino-acid LFLAK motif [13]. The majority (37) of coding sequences lacked both 5′ and 3′ sequences. Twenty lacked 5′ sequences while four lacked a stop codon and apparently lacked 3′ sequences. Three sequences were complete, but only one (PcCRN2) were identified with a predicted signal peptide (Additional file 11). *PcCRN2* was previously detected in a transcriptional study of *P. cactorum* (named T511E4 therein) where it was found up-regulated during cyst germination and host infection [8].

**Figure 4:**
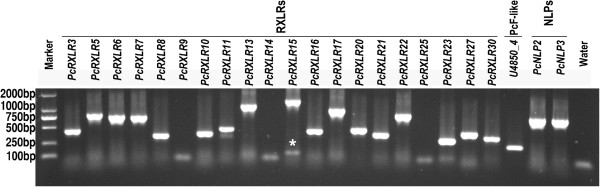
**PCR amplification of a subset of the 23 predicted** ***P. cactorum*** **unigenes.** PCR amplification was performed on *P. cactorum* isolate 10300 genomic DNA with 23 primer pairs, and the amplicons were electrophoresed on an agarose gel (1.2%). Lane Marker, DL-2000 DNA marker ladder (TaKaRa, Dalian, China). Gene names and categories are listed on the top of the gel picture. The star indicates a non-specific amplification band for *PcRXLR15*. Lane Water, water control with *PcNLP3* primers (Additional file 12).

The number of “real” effector genes in our dataset is yet to be determined although obvious pseudogenes were omitted. However, some of the predicted effector genes were validated in this study. Twenty-three effector sequences including 20 RXLRs, 2 NLPs and one PcF-like were selected to check the accuracy of the computational analysis (Figure 4 and Additional file 12). They were tested by PCR amplification using *P. cactorum* genomic DNA. A total of 19 amplifications produced a unique band of the expected size (Figure 4). One sequence, *PcRXLR15*, produced two amplified bands, but the brighter one exhibited the expected size, suggesting the lower band resulted from non-specific amplification. Three primer pairs towards the RXLR candidate genes (*PcRXLR10*, *PcRXLR14* and *PcRXLR30*) did not produce expected bands (Figure 4). These 20 genes were then full-length cloned and sequenced, yielding the same sequences as transcriptome assembly data. Due to the approximately 87% correct predictions (20/23), the *in silico* analysis in this study was considered as providing reliable results.

### The WY-domain is conserved in about half of *P. cactorum*RXLR effectors

Recently, it was identified by structural biology that WY-domain is a structure unit conserved in many *Phytophthora* and *H. arabidopsidis* RXLR effectors [38, 39]. We searched *P. cactorum* RXLR effector candidates using the HMM for the WY-domain as described previously [38]. Using the score cut-off of 0.0, we revealed that 45 of 94 (48%) of *P. cactorum* RXLR effector candidates contain WY-domain-like sequences (Additional file 9 & Table 4). WY-domains can also be found in tandem repeats, and we identified 21 candidate effectors with more than one WY-domain. Two candidate effectors, PcRXLR15 and PcRXLR59, contain 5 WY-domains. Therefore, many *P. cactorum* RXLR effector candidates comprise either single or tandem arrays of WY-domains.

### The expression of most effector genes is induced during *P. cactorum*infection

Our study showed that *P. cactorum* can infect the model plant *N. benthamiana* (Figure 2). No evident macroscopic symptoms were observed in the root until 48 hpi (root surface browning). The disease progressed quickly in the plants, and by 4 d post-inoculation all inoculated plants were wilting or dead (Figure 2B). To investigate the effector gene expression pattern changes during host infection, the infected *N. benthamiana* roots were harvested at 7 infection time-points (1.5, 3, 6, 12, 24, 48 and 96 hpi). This assured that the whole infection period of *P. cactorum* on *N. benthamiana* could be investigated. cDNA samples from four life stages (MY, SP, ZO and GC) were also obtained as described previously but with one modification in which here GC was induced directly on *N. benthamiana* leaves. Hence GC sample used here is a mixture of microbe and plant materials. Expression experiments were performed for 30 effector genes including 28 RXLRs, one elicitin (unigene ID *U7626_1*, hereafter referred to as *PcELL1*) and one NLP (unigene ID *U2101_5*, hereafter referred to as *PcNLP1*). Those 28 representative RXLR unigenes were chosen based on a consideration of the signal peptide confidence, the intact ORF and RXLR-dEER presence. The transcript levels in the 11 RNA samples were assessed using semi-quantitative RT-PCR.

**Figure 5:**
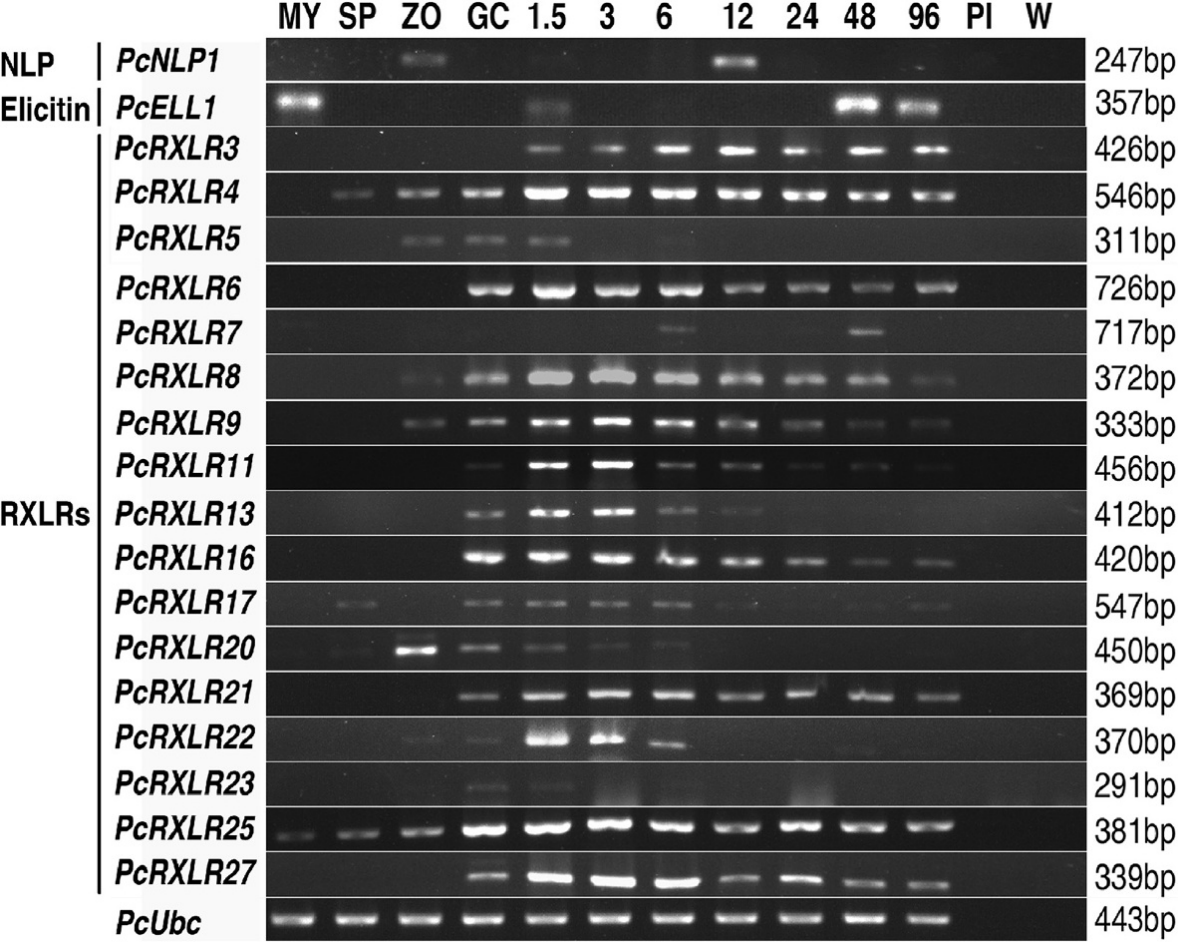
**Expression patterns of 19** ***P. cactorum*** **effector genes during life cycle and infection stages.** Lanes: MY, plate-grown mycelia; SP, sporangia; ZO, zoospores; GC, germinating cysts directly on *N. benthamiana*; 1.5 - 96, *N. benthamiana* roots at 1.5, 3, 6, 12, 24, 48, 96 hours post-inoculation (hpi); Pl, mock-inoculated plants; W, control leaves inoculated with distilled water. Gene names and categories are listed on the left and the amplicon sizes are listed on the right. All sizes were as expected, and the experiment was performed with at least three biological replicates.

Eleven of the designed RXLR primer pairs produced unspecific (7) or no (4) fragments for the infection samples, and were not included for further analyses. Figure 5 shows the expression patterns obtained for the remaining 17 RXLR and 2 other genes. All these RXLR genes were up-regulated during the host invasion, relative to MY that can be regarded as the vegetative growth stage. In particular, all but one RXLR gene (*PcRXLR7*) were highly expressed during the earliest infection stages (1.5 hpi). Of these 16 early expressed genes, 14 also showed induced expression at GC which is an essential pre-infection stage of *P. cactorum*. The differential expression of 6 RXLR candidate genes (*PcRXLR6*, *PcRXLR8*, *PcRXLR13*, *PcRXLR16*, *PcRXLR21* and *PcRXLR25*) in comparison between GC and MY was further validated by qRT-PCR and RPKM analyses (Additional file 1). Two RXLR genes (*PcRXLR11* and *PcRXLR4*) were previously detected in a transcriptional study of *P. cactorum* (named *T511H3* and *T512B6*, respectively) where they were expressed during strawberry infection and cyst germination [8]. That result is consistent with the result obtained in this study.

In addition, the expression of two other effector genes was examined (Figure 5). The elicitin gene *PcELL1* showed stronger expression both at MY and during late infection of *N. benthamiana* (48 and 96 hpi) versus at 1.5 hpi. The NLP gene *PcNLP1*, however, showed expression only at ZO and 12 hpi.

### *P. cactorum*effectors elicit plant cell death and are conserved across oomycete species

**Figure 6:**
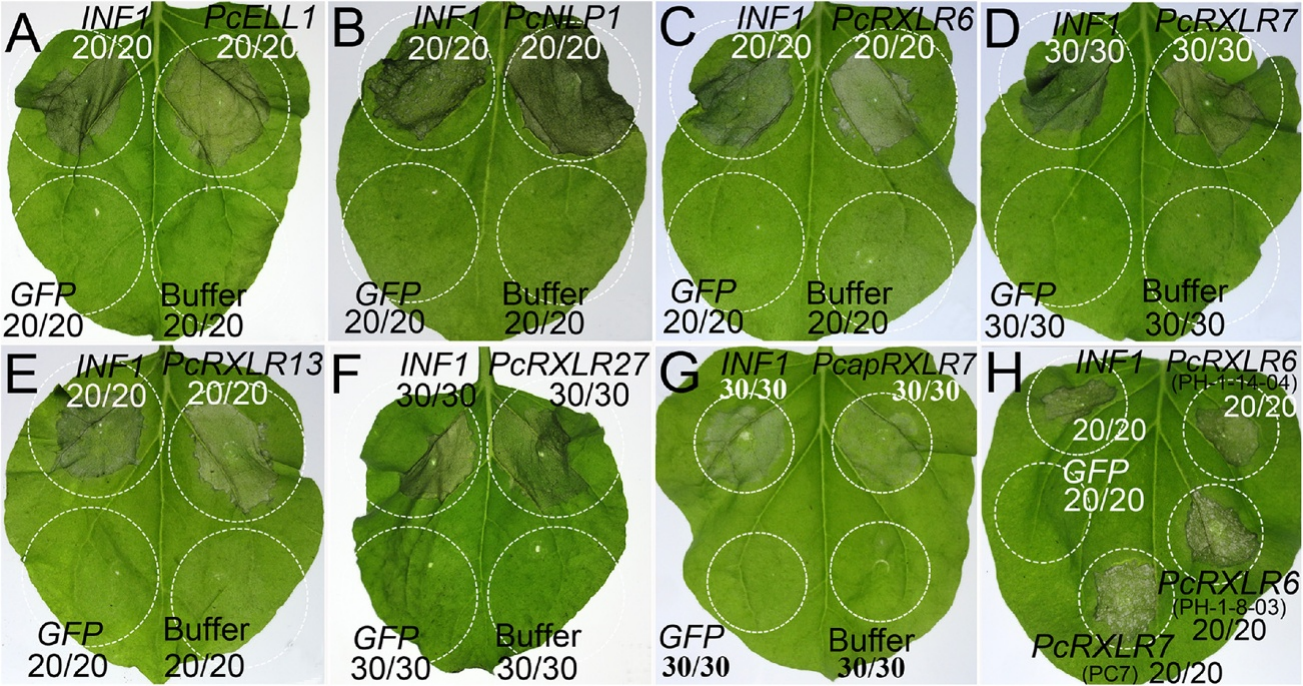
**Transient assays of** ***Phytophthora*** **RXLR, NLP and elicitin effectors in** ***N. benthamiana*** **.** The *P. cactorum* elicitin PcELL1 **(A)**, NLP PcNLP1 **(B)**, four RXLRs (PcRXLR6, PcRXLR7, PcRXLR13 and PcRXLR27) [**(C)**-**(F)** in order], *P. capsici* RXLR PcapRXLR7 **(G)** and the alleles of PcRXLR6 and PcRXLR7 **(H)** triggered plant cell death. The plant leaves were infiltrated with *A. tumefaciens* (strain GV3101) cells to express each of these effectors. Agro-infiltration sites in each *N. benthamiana l*eaf expressing *INF1* (top left) were the positive controls. The other sites in leaves expressing *GFP* only, or infiltrated by 10 mM MgCl_2_ buffer only served as negative controls. *PcapRXLR7* is the ortholog gene of *PcRXLR7* in *P. capsici*. The alleles of *PcRXLR6* in the isolates PH-1-14-04 and PH-1-8-03 are labeled as *PcRXLR6* (PH-1-14-04) and *PcRXLR6* (PH-1-8-03), respectively while the allele of *PcRXLR7* in PC7 as *PcRXLR7* (PC7). Photographs were taken 5 [**(A)**-**(C)**, **(E)**-**(F)** and **(H)**] or 7 [**(D)**, **(G)**] d after infiltration. The circles indicated the agroinfiltration areas.

Expression studies indicated that we had identified infection-related effector genes in *P. cactorum*. To begin an investigation of the activity of those potential effectors *in planta*, we conducted heterologous expression assays in *N. benthamiana* using infiltration of *A. tumefaciens* cells with the vector pGR107 (Figure 6). These methods have been proved valuable in the characterization and determination of the function of a variety of effector genes [9, 13, 40, 59–62]. All of the 19 aforementioned effectror genes were cloned into the transient expression vector pGR107. A known elicitor of cell death, the *P. infestans* PAMP INF1 [63], was used as positive control. *A. tumefaciens* strains harboring *GFP* served as negative control. The elicitin PcELL1 (Figure 6A), NLP effector PcNLP1 (Figure 6B) and four RXLR effectors (PcRXLR6, PcRXLR7, PcRXLR13 and PcRXLR27) (Figure 6C-F) triggered leaf cell death in *N. benthamiana* as vigorously as INF1 (Figure 6). Expression of *PcRXLR27*, *PcELL1* and *PcNLP1* resulted in leaf chlorosis throughout the entire infiltration zone by 4 dpi, followed by initiation of cell death at 5 dpi. By 7 dpi, the zone of infiltration was completely dehydrated. The cell death symptoms triggered by PcRXLR13 and PcRXLR6 developed about 5 d after infiltration whereas the symptoms caused by PcRXLR7 developed about 7 d after infiltration. In contrast, only mosaic symptoms due to PVX were observed following infiltration with the remaining 13 RXLR effector genes or with *GFP* under the same conditions.

We performed multiple sequence alignment of these elicitors of cell death, i.e. four RXLR effectors (PcRXLR6, PcRXLR7, PcRXLR13 and PcRXLR27), PcELL1 and PcNLP1 to determine their conservation within oomycetes (Additional file 13). Based on the conserved features and domain organization of oomycete effectors, the presence of a canonical RXLR motif and an EER motif was identified at N-termini of *P. cactorum* RXLR effectors and their orthologs in other *Phytophthora* species (Additional file 13). BLASTx results showed that *P. cactorum* PcRXLR6, PcRXLR7, PcRXLR13 and PcRXLR27 have 34%, 73%, 32% and 41% identity to *P. infestans* RXLR homologues, respectively. In contrast, these four effectors displayed higher sequence identity (49%, 75%, 62% and 51%, respectively) to *P. parasitica* homologues. However, the *P. parasitica* homologues of PcRXLR6 and PcRXLR13 were annotated as hypothetical proteins in the INRA-310 (v2) reference genome (Additional file 10) although the canonical RXLR-EER motif is present (Additional file 13). Except PcRXLR6, the other three RXLR effectors have homologues in *P. capsici* and *P. sojae* (Additional file 13). Noticeably, PcRXLR13 and PcRXLR27 showed high sequence similarity (62%, 71%) to *P. sojae* effectors Avh147 and Avh238, respectively, which have been identified to trigger cell death in *N. benthamiana* and soybean [62]. As reported previously [56], the RXLR motif of PcRXLR6, PcRXLR13, PcRXLR27 and their orthologs in other *Phytophthora* species is confined to position 30–60 amino acids (Additional file 13). However, analysis methods employing this constraint most likely eliminate genuine effectors. In this study, the canonical RXLR motif is located between residues 30 and 100 in the amino acid sequences of PcRXLR7 and its orthologs (Additional file 13). Data mining revealed that the coding sequence of its *P. capsici* ortholog, hereafter referred to as *P. capsici* RXLR protein 7 (*PcapRXLR7*), is embedded in a cDNA clone CBOT37-C15 [GenBank: BT031570] but not annotated in the *P. capsici* reference genome (scaffold_189084054). We cloned *PcapRXLR7* into pGR107 and performed transient assay as described previously. Intriguingly, PcapRXLR7 also triggered cell death in *N. benthamiana* leaves about 7 d after infiltration (Figure 6G). The *P. capsici* orthologs of PcRXLR13 and PcRXLR27 did not induce PCD in *N. benthamiana*. The above results together suggest that these RXLR effector proteins from different *Phytophthora* pathogen species trigger similar responses in plant families.

One NLP effector of interest, PcNLP1, showed 110 of 256 amino acids conserved across four different *Phytophthora* species and an additional oomycete, *Pythium ultimum*, whereas 219 were shared with *P. parasitica*, corresponding to 85% identity (Additional file 13). The data showed that all aligned NLPs contained two conserved cysteine residues which defined these proteins as type I NLP [19]. We also noted that the central conserved GHRHDWE heptapeptide motif is present in all NLPs (Additional file 13). The elicitin PcELL1 shared 77 amino acids with four *Phytophthora* species and *Py. ultimum*, whereas 107 of 118 amino acids (91%) were conserved between *P. cactorum* and *P. infestans* (Additional file 13).

### Polymorphic sites in *P. cactorum*RXLR effector sequences

**Figure 7:**
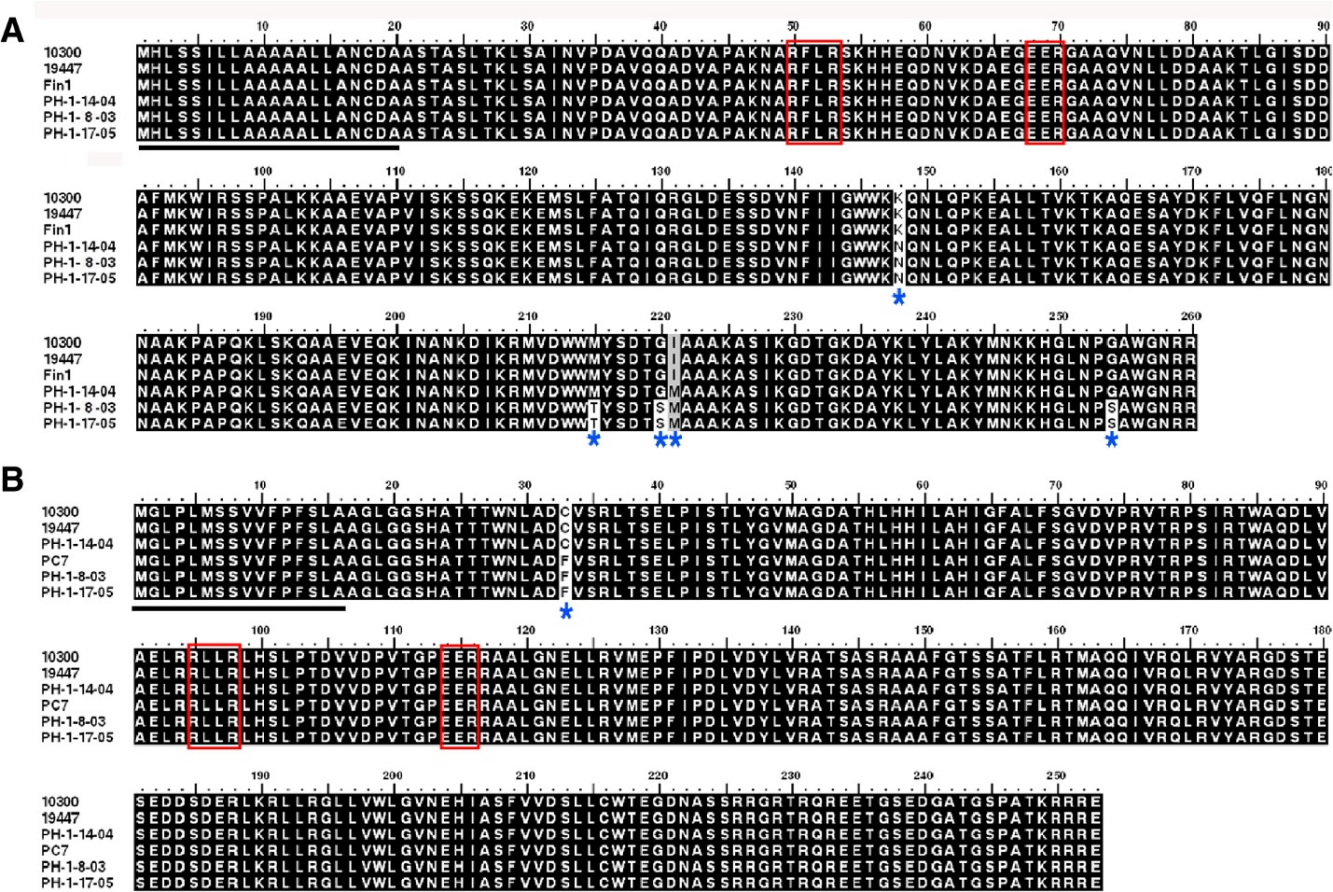
**Sequence polymorphisms of RXLR effector alleles among** ***P. cactorum*** **isolates. (A)** The protein sequences of PcRXLR6 in six isolates (10300, 19447, Fin1, PH-1-14-04, PH-1-8-03, PH-1-17-05) were compared. The allele was not obtained from the isolate PC7. **(B)** The protein sequences of PCRXLR7 in six isolates (10300, 19447, PC7, PH-1-14-04, PH-1-8-03, PH-1-17-05) were compared. The allele was not obtained from the isolate Fin1. The putative RXLR and EER motifs of both effectors were denoted by red boxes. Their predicted signal peptide sequences were underlined. The polymorphic sites were marked by blue asterisks for the visualization.

The four RXLR genes that triggered PCD were examined in a collection of seven *P. cactorum* isolates (Additional file 14), to determine whether sequence polymorphisms may exist in these genes. Effector genes may exhibit a high level of polymorphism if they are subject to diversifying selection as a result of pressure from plant resistance genes. Two (PcRXLR6 and PcRXLR7) of the four RXLR genes were found to exhibit non-synonymous polymorphisms that altered the amino acid sequences they encoded (Figure 7). Variations in 5 amino acid residues were identified in PcRXLR6 among the 6 sequenced isolates (Figure 7A). These 5 residues were located at the C-terminus of the sequence. The same fragment was not amplified from the isolate PC7 by PCR, suggesting that the gene may be deleted or substantially altered in this isolate. Noticeably, the effector sequence was identical among the three European strawberry isolates, and in the two Persian walnut isolates. PcRXLR7 could be amplified from all strains but Fin1, but showed only one polymorphic site at the N-terminus among those six isolates (Figure 7B). In contrast, PcRXLR27 and PcRXLR13 showed no amino acid polymorphisms among the 7 tested isolates.

The ability of the different effector alleles to trigger PCD was tested (Figure 6H). The alleles of PcRXLR6 amplified from the isolates PH-1-8-03 and PH-1-14-04 induced the same PCD as the one from the isolate 10300 did (Figure 6C and H). Similarly, PcRXLR7 allele in PC7 also produced the same symptom on plants as PcRXLR7 of the isolate 10300 did (Figure 6D and H). The results indicated that the polymorphic sites in these RXLR effectors do not affect their ability to trigger plant cell death.

We further examined the strength of natural selection acting upon the effectors (PcRXLR6 and PcRXLR7) by estimating the ratio of the non-synonymous substitution rate (d*N*) to the synonymous substitution rate (d*S*) (Table 5). We defined the boundary of the N (including RXLR and EER) and C termini of protein sequences as the 70th (PcRXLR6) or 116th (PcRXLR7) amino acid (Figure 7). The Yn00 tool in the PAML v4.7 software package was used to calculate the ratio of d*N* to d*S*[64]. Three representative sequences made up three pairwise comparisons for the test of PcRXLR6. There is no polymorphism in the N-terminus of PcRXLR6 but in the C-terminus. However, no significant evidence of selection pressure in the C-terminus of PcRXLR6 was identified though the pairs 10300/PH-1-8-03, PH-1-8-03/PH-1-14-04 displayed dN > dS and the pair 10300/PH-1-14-04 showed dN < dS. Single polymorphism site was found in the N-terminus among alleles of PcRXLR7. Although dN > dS, it may not be significant (Table 5). Consistent with these results, model comparisons using the computer program CODEML [64] did not reveal any positively selected sites for either of the RXLR effectors.

**Table 5:** **d** ***N*** **/d** ***S*** **analysis of pairwise comparisons of the RXLR alleles**

Gene ID	Pairwise comparisons		Full sequences				N-terminus ^b^				C-terminus ^b^			
	Seq. 1	Seq. 2	d ***N***	d ***S***	d ***N*** /d ***S*** ^a^	***P*** value	d ***N***	d ***S***	d ***N*** /d ***S*** ^a^	***P*** value	d ***N***	d ***S***	d ***N*** /d ***S*** ^a^	***P*** value
*PcRXLR6*	10300	PH-1-8-03	0.0091	0.0045	2.0222	4.5e-01	0	0	–	7.3e-01	0.0122	0.0064	1.9063	5.8e-01
	10300	PH-1-14-04	0.0036	0.0089	0.4045	4.4e-01	0	0	–	5.9e-01	0.0049	0.0128	0.3828	5.4e-01
	PH-1-8-03	PH-1-14-04	0.0054	0.0045	1.2000	8.7e-01	0	0	–	9.2e-01	0.0073	0.0064	1.1406	8.7e-01
*PcRXLR7*	PC7	10300	0.0019	0	∞	3.2e-01	0.0042	0	∞	3.2e-01	0	0	–	6.0e-01

^a^The ‘∞’ in column d*N*/d*S* refers to pairwise comparisons in which d*N* >0 and d*S* =0, and ‘–’ refers to pairwise comparisons in which d*N* =0 and d*S* = 0.

^b^The N-terminus refers to the sequence from the 1st to the end of dEER, and the C-terminus refers to the sequence from the immediate residue after dEER to the end, except the stop codon.

## Discussion

Despite the worldwide economic impact of diseases caused by *P. cactorum*, little is known about the molecular basis of the pathogenicity of this species. The availability of the genome sequences from other pathogens and their host plants has greatly advanced our understanding of pathogen virulence and host resistance [65, 66]. Davis and Yu [67] and Shulaev et al. [68] presented the first genetic map and a genome sequence, respectively, for woodland strawberry (*Fragaria vesca*), which is one important host of *P. cactorum*. In contrast, a lag still exists in developing these resources for the hemibiotrophic pathogen *P. cactorum*. Chen et al. [8] provided a first overview of genes that may be involved in *P. cactorum* pathogenicity. However, the sequence data obtained in that study were limited. Additionally, due to the short sequences and lack of a reference genome, cloning strategies such as rapid-amplification of cDNA end (RACE) had to be employed to get the full length genes for ESTs of interest [8]. The sequence data created in this study therefore represent a substantial expansion of the transcriptome resources available for *P. cactorum* and a glimpse into its pathogenicity mechanisms.

Due to the common problem that contamination of plant material in infection-stage samples often makes it difficult to study the plant-microbe interactions [8, 69], examining *Phytophthora* life cycle stages such as germinating cysts may be a useful alternative for investigating the transcriptional changes of infection-related *Phytophthora* genes. Previous studies have shown that molecules involved in establishment of infection and elicitation of plant defenses are extensively expressed during the life cycle stages [4–9]. Hence, in the present study the *P. cactorum* transcriptome sequences were generated by Illumina sequencing from five successive life stages (MY, SP, ZO, CY and GC), and assembled by *de novo* short read. To prepare the important pre-infection stage, the germinating cysts (GC), a cellophane membrane was placed on *N. benthamiana* leaf to mimic natural infection and induce germination of cysts while minimizing contamination of plant material. Microscopic analysis first demonstrated that germinating cysts on cellophane morphologically resemble the ones germinating directly on *N. benthamiana* leaves, in contrast to the ones in water (Figure 1E-G). Second, the gene expression pattern in cysts germinating under both mimicry and natural conditions was investigated and compared. In GC versus MY, the RXLR candidate genes (*PcRXLR6*, *PcRXLR8*, *PcRXLR13*, *PcRXLR16*, *PcRXLR21* and *PcRXLR25*) under mimic condition showed the same expression pattern as during natural infection (Additional file 1 and Figure 5). The direct comparison provided further justification that our mimic experiments closely mirror the infection process occurred in fields. Thus, cellophane membrane was a valid model for the cyst germination. The *in vitro* inoculation method could be applied to other similar plant-pathogen interaction studies.

Using the CEGMA tool, 243 (98%) of the 248 conserved single-copy eukaryotic genes mapped against the transcriptome assembly were identified in our study. Similarly, 93.6% and 95% of 248 CEGMA genes were reportedly detected in *A. laibachii* and *H. arabidopsidis* genome assemblies, respectively [50, 70]. The high recovery of 98% of CEGMA genes suggests a high quality *P. cactorum* transcriptome assembly in the present study. Comparisons to Sanger-derived sequences from *P. cactorum* (Additional files 4 and 5), and PCR validation (Figure 4) further showed that the transcript assemblies are robust. Comparison of assembled gene models (21,662) to gene catalogs of other oomycete species by BLASTx analysis and functional annotation (e.g., TribeMCL, GO, COG and KEGG) indicate that the sequences represent an extensive catalog encompassing a large proportion of the genes expressed in *P. cactorum*. In the study, we used the data from TribeMCL to globally examine the conservation of *P. cactorum* genes across *Phytophthora* species (Figure 3). Overall, 5,538 gene families contained sequences from all four species, representing about 65% (5,538/8,582) of the total number of gene families identified in this study. Of that, 365 gene families were specific for *P. cactorum* and *P. infestans*. In contrast, 94 gene families were specific for *P. cactorum* and *P. sojae*, and 40 for *P. cactorum* and *P. ramorum*. This fits well with the fact that *P. cactorum* lies more closely with *P. infestans* than with others in the phylogenetic tree [57]. Among those 2,471 putative *P. cactorum* proteins that lack homologs in other species, 85 were grouped into 36 paralogous groups (Figure 3). The remaining 2,386 were considered singletons, as they lack homologs within their own proteome or in the other taxa. This number is a bit larger than the counterpart numbers from each of other three species (863 singletons for *P. infestans*, 633 for *P. sojae* and 113 for *P. ramorum*). This could be due to the sequencing depth and therefore assembly in this study. The assembly quality of these sequences could be improved in the future by even deeper sequencing and by genome sequencing. Alternatively, these proteins could potentially represent *P. cactorum*-specific orphan genes. However, more research is required to dissect this. On the basis of gene annotations and pathway analyses, the *P. cactorum* unigenes are predominantly involved in cellular and metabolic processes, the binding and catalytic activities and cellular components (Additional files 6 and 7). Similar results were found in *P. infestans* (Additional file 6). In addition, overall 2,717 unique transcripts were predicted to be involved in 275 KEGG metabolic pathways, with two major pathways (ribosome and chromosome) comprised of over 450 unigenes (Additional file 8). The predicted pathways together with the gene annotations will be useful for further investigations of gene function in the future.

Potential apoplastic and host-translocated effectors could be predicted from the assembled transcript sequences. In the present study, 620 *P. cactorum* genes were identified encoding transporter or effector proteins with putative roles in virulence (Table 3), with a repertoire similar to other oomycete plant pathogens. The *P. cactorum* transcripts obtained here represent the genes expressed during important life stages, and may therefore not represent all genes that are present in the genome. The *P. cactorum* effector data set could be expanded by including additional life cycle and infection stages of *P. cactorum*.

Sequencing of the genomes of *Phytophthora* plant pathogens has revealed a variable number of RXLR effectors in different species [12, 13]. We identified 94 potential RXLR effectors in the *P. cactorum* transcriptome, 46 of which contained a conserved RXLR motif (Additional file 9 and Table 4). Additionally, 10 potential effectors contained a variant of RXLR motif (RXLX) that is similar to the Pexel translocation motif of *Pl. falciparum* effectors (RXLX^E^/Q_/D_). *Plasmodium* Pexel domains can functionally replace the RXLR-dEER region of Avr1b from *P. sojae* and *P. infestans* Avr3a [25, 71]. Furthermore, mutation of the fourth position of the *P. sojae* RXLR motif did not affect translocation [26]. Of the 94 *P. cactorum* RXLR effector candidates, 43 sequences appeared to be complete (Additional file 9 and Table 4). We examined 28 representative effectors (all with a canonical RXLR-dEER motif) using both RT-PCR (Figure 5) and PCR cloning (Additional file 12). Seventeen full length sequences were cloned and confirmed to be RXLR genes. The other 11 sequences were not further analyzed due to unspecific (7) or no (4) bands amplified from infection-stage samples in RT-PCR assays. Among these 4 sequences, only one can not produce any bands from all the test samples (including non-infection samples). More specific primers towards these 11 sequences should be designed to dissect this. And, more work is needed to determine the number of functional or “real” genes in this dataset though obvious pseudogenes were excluded. Two RXLR genes (*PcRXLR11* and *PcRXLR4*) out of 11 RXLR gene candidates reported in a previous study [8] (named *T511H3* and *T512B6* therein, respectively) were re-detected in this study. The others mostly discovered by effector-specific differential display (ESDD) technique [8] have not been re-detected in this study. However, this is not surprising because effector genes could be differentially expressed at different stages in response to different conditions. The 94 RXLR effector candidates were detected by RNA-seq during the pathogen developmental stages in the present study while most (8) of 11 potential RXLR effectors were identified during the strawberry infection by ESDD in the previous study [8]. Some effector genes could be stage-specific and therefore not detected in both cases.

The C-terminal regions of oomycete RXLR effectors carry the biochemical effector activity and about half of these proteins share a conserved WY-domain [38, 39]. This WY-domain fold may be an adaptive structural unit that can support effector diversification to gain new functions and/or evade plant host immunity [39]. Using HMM–based sequence searches, WY-domain had also been detected in our *P. cactorum* RXLR effector candidates, with 45 out of 94 (48%) containing this fold (HMM score > 0) (Additional file 9). This strengthens that the WY-domain unit could be critical for the success of oomycete plant pathogens. However, the reason why the WY-domain is preserved and its contribution to the functions of the effectors are still unknown. Future studies are required to help define the roles of the WY-domain fold in the virulence mechanisms of these pathogens.

We examined the transcript levels of those 28 representative effector genes by hemi-quantitative RT-PCR and observed high expression during the host infection for 17 of them (Figure 5). Nearly half of the RXLR genes were differentially expressed during the life cycle and early infection stages of the *P. cactorum*. The expression patterns of the RXLR effector genes are consistent with their expected roles in assisting the colonization of plant tissues by manipulation of host defense responses [59, 60]. To explore their predicted roles further, we tested if the RXLR effectors could cause phenotypic reactions when expressed in host plants and found that four effectors could elicit plant cell death (Figure 6). Effectors from other oomycete plant pathogens, including Avh147, Avh238, Avh241 from *P. sojae*, PcapRXLR7 from *P. capsici* (Figure 6G), PscRXLR1 and PcQNE from *Pseudoperonospora cubensis* and members of the CRN family from *P. infestans* and *P. sojae*, have also been shown to elicit similar phenotypes when transiently expressed in *N. benthamiana*[29, 40, 43, 44, 72, 73]. The counter-intuitive ability of oomycete effectors that are expressed during biotrophy to trigger cell death has been inferred to reflect the ability of the effectors to manipulate the plant defense responses and/or the ability of the plant defense machinery to initiate effector-triggered immunity. Wang et al. [62] showed that a minority of *P. sojae* RxLR effectors (including PcRXLR13 and PcRXLR27 homologues, Avh147 and Avh238, respectively) triggered cell death in the host, soybean, or in *N. benthamiana*, and that a majority of tested *P. sojae* effectors could suppress the cell death triggered by those effectors. Thus during natural infection it is likely that such effectors do not actually induce cell death, but instead manipulate the plant immune system in other ways that promote infection.

Previous studies have shown how the primary sequences of many RXLR effectors have been shaped by positive selection, and how the genes themselves undergo accelerated birth and death evolution [31, 35, 36, 56, 74]. Two of the cell death-inducing effectors, PcRXLR6 and PcRXLR7, exhibited polymorphisms but no significant evidence of natural selection among seven *P. cactorum* isolates (Figure 7 and Table 5). The polymorphisms were localized basically to the C-terminal effector domains. Generally the amino acid sequences of each of both RXLR effectors were identical within isolates from the same host while different among isolates from different hosts. These host-related patterns may have been molded by host preferences, geographic isolation and/or degree of specialization, since the RXLR secretome is at the front line in the evolution of the host-pathogen interaction [35, 56]. The polymorphic nature of these *P. cactorum* RXLR effectors reinforces that these genes may play an important role in the microbe-plant interaction. However, no residues under positive selection in these two RXLR effectors were identified. This is possibly because only seven *P. cactorum* isolates were investigated for sequence polymorphism. The alleles of PcRXLR6 and PcRXLR7 retain PCD-triggering activity although their sequences showed polymorphism. The *P. cactorum* PCD-inducing RXLR effectors are highly conserved across several *Phytophthora* species (Additional file 13). Ectopic expression data showed that the *P. capsici* ortholog of PcRXLR7 (PcapRXLR7) (Figure 6G) and *P. sojae* orthologs of PcRXLR13 and PcRXLR27 (Avh147 and Avh238, respectively) [62] also triggered PCD *in N. benthamiana*, emphasizing their importance. These PCD-inducing effectors maybe represent few RXLR effectors conserved across *Phytophthora* species [35]. Whether such RXLR ortholog pair targets a common plant component needs further investigations.

In this study, the genes for the elicitin PcELL1 and the NLP PcNLP1 were also validated, cloned and transiently expressed in plants. Both genes showed distinct expression during the developmental and infection stages (Figure 5). Ectopic expression in *N. benthamiana* displayed that both genes can trigger plant cell death (Figure 6A-B). Recent studies on other *Phytophthora* species revealed similar results [9, 15, 16, 75]. Based on multiple sequence alignment, we found that these effectors are highly conserved across oomycete species (Additional file 13). Together, these results suggested their important roles in the pathogenicity of *P. cactorum.* Future work should determine the number of functional or “real” genes encoding these effectors in *P. cactorum* and elucidate their functional roles in the pathogen’s virulence.

## Conclusions

As a root pathogen, and because of its broad host range, *P. cactorum* is an attractive model for understanding *Phytophthora* infection. Sequencing of the *P. cactorum* transcriptome has provided a wealth of information about the proteins expressed during the important life stages, especially the RXLR effectors. This is the first publication using next generation sequencing technology for *P. cactorum* without genome information. We have demonstrated the reliability of the transcriptome assembly by CEGMA, PCR validation and (q)RT-PCR analyses. A large number of genes including 94 RXLR effector candidates and gene families of *P. cactorum* were identified. About half of the RXLR effector candidates are predicted to have a similar overall conformation, termed the WY-domain fold. Additionally, we have demonstrated the identification and characterization of PCD-triggering RXLR, NLP and elicitin effectors. More studies will however be required to determine the roles of infection-related effector genes in the pathogenesis. The results will ultimately help to elucidate the mechanisms underlying the pathogenicity of *P. cactorum* and to design effective control strategies for the diseases caused by this broad host range pathogen.

## Methods

### Oomycete materials

*P. cactorum* strains (Additional file 14) were routinely cultured on 10% V8 agar media at 25°C in the dark [1]. Strain 10300 was used for all the experiments except for the polymorphism analysis in which all 7 isolates were employed.

For nucleic acid extraction, mycelia were cultivated in 10% V8 broth media at 25°C in darkness for 96 h, then blotted dry with absorbent paper and preserved in liquid nitrogen for further applications. Sporangia and zoospores were prepared as previously described [8]. Cysts were induced by vigorously vortexing the zoospore suspension for 30 s. To obtain germinating cysts, a solution containing approximately 1000 encysted zoospores was dropped onto a pre-treated cellophane membrane placed on a detached *N. benthamiana* leaf [53]. A moist Whatman filter paper was already placed underneath the leaf in a sterile Petri dish and the leaf was incubated at 25°C for 1–2 h in the dark. This procedure mimics cyst germination on a host surface but avoids the inclusion of plant tissue. Cyst germination was regularly observed every 10 min under a light microscope after 45 min post-inoculation. Germinating cysts were collected when the average length of germ tubes of 80% germinating cysts was twice the length of the average diameter of cysts. Sporangia, zoospores, cysts or germinating cysts were collected by centrifugation at 1,500 × *g* for 10 min and snap frozen in liquid nitrogen for RNA isolation.

### Plant inoculation and trypan blue staining

*N. benthamiana* plants were cultivated in Styrofoam cups containing sterile soil and placed in the greenhouse at 25°C with 16/8-h light–dark photoperiod, until 5 to 6 weeks old.

Inoculation of *N. benthamiana* leaves with *P. cactorum* was conducted similarly as described [76] with the following minor modifications. Detached leaves of uniform size and age placed on moist filter paper in Petri dishes with the adaxial or abaxial surface upward (experiments were done both ways). A zoospore suspension (50 μl; approximately 1 × 10^5^ zoospores/ml) was placed onto the right side of the main vein, while sterile water was placed onto the left side as a control. The leaves were incubated at 25°C in darkness for varying times (3, 6, 12, 24 hpi). Trypan blue staining was performed to detect cell death in the *N. benthamiana* leaves. The leaves were soaked in Farmer’s solution (95% ethanol: chloroform: glacial acetic acid, 6:3:1) for 30 s and then submerged in a 0.05% trypan blue mixture (Sigma-Aldrich, St. Louis, MO) for 8 h. The leaves were rinsed with deionized water and then destained using multiple changes of boiling 95% ethanol. The infected areas of leaves were then compared with the mock-inoculated controls and photographed.

For root inoculation, plants were watered 2 to 3 h prior to inoculation to ensure that the soil was wet. Each plant was inoculated around the roots with 9 ml of a freshly prepared zoospore suspension. Plants mock-inoculated with sterile water were used as controls. All the plants were incubated in a growth chamber offset to 25 ± 1°C, a relative humidity of 98% ±1% and a cycle of 16 h light and 8 h night. The infected portions of the inoculated root were harvested at 1.5, 3, 6, 12, 24, 48, 96 hpi for gene expression analyses. No evident macroscopic symptoms were observed in the root until 48 hpi (Figure 2); so for the earlier time points, the portion of the root that had been in direct contact with the inoculum was harvested. To monitor the success of the inoculations, additional inoculated and mock-inoculated plants were kept for 9 days after inoculation for continuous observation. All samples were snap frozen in liquid nitrogen and stored at – 75°C until RNA extraction.

### Light and Cryoscanning electron microscopy

For light microscopy, an Olympus System Microscope BX53 (Olympus Corporation, Tokyo, Japan) was used, and an Olympus DP72 digital camera for photography.

Samples with high water content could not be directly observed using scanning electron microscope (SEM). Cryofixation was used to avoid artifacts produced by chemical fixation and critical point drying. To observe cysts germinating directly on *N. benthamiana* leaves, inoculation sites were sampled by excising 5-mm square leaf segments. Leaf segments, three at a time, were loaded on the cryo-specimen holder and cryo-fixed in nitrogen (− 210°C), then quickly transferred to the cryo-unit in the frozen state. The segments were sublimed at – 85°C in a vacuum SEM chamber and then examined in the cryo-stage of an S-4800 SEM (Hitachi High-Technologies Corp, Tokyo, Japan) with a temperature controller under an accelerating voltage of 15 keV. Using this CryoSEM technique, the inoculation surface of the frozen leaf segments was viewed directly while being maintained at – 135°C. Imaging was performed by collecting the back-scattered electron (BSE) signal with a sensitive crystal detector.

### Isolation of nucleic acids

Genomic DNA was isolated from *Phytophthora* mycelia as described [77]. Total RNA was extracted from samples using RNAiso Plus reagents (Takara Biotechnology [Dalian], China) according to the manufacturer’s instructions. The quality and quantity of both DNA and RNA were checked by gel electrophoresis and spectrophotometrically. Before cDNA synthesis, RNA samples were treated with DNase I (TaKaRa) to eliminate trace genomic DNA. PCR (45 cycles without reverse transcriptase) was used to check that RNA samples were free of genomic DNA. All RNA samples were stored at – 75°C until use.

### mRNA-Seq library construction and sequencing

Five sources of RNA were used for *P. cactorum* transcriptome sequencing: RNAs isolated from mycelia (MY), sporangia (SP), zoospores (ZO), cysts (CY) and germinating cysts (GC). Equal amounts of total RNA from each source were pooled for Illumina mRNA-Seq. Two independent biological replicates were sequenced in parallel. The pooled RNA was used to prepare libraries following the manufacturer’s instructions (Illumina, San Diego, CA, USA), and paired-ends were sequenced on an Illumina Genome Analyzer IIx for 75 or 83 cycles for Library 1 and Library 2, respectively (Additional file 2).

### Transcriptome assembly

Raw paired-reads from the two replicates were combined for sequence cleaning. Adapter sequences, low-quality sequences with ambiguous bases, and reads with more than 10% Q <20 bases were filtered out. After trimming, sequences shorter than 60 bp and those with a “G + C” content outside 20%-80% were excluded. The filtered and trimmed reads were used for downstream analysis. Transcriptome *de novo* assembly was carried out with Trinity software [78] that can recover full-length transcripts across a broad range of expression levels, with sensitivity similar to methods that rely on reference genome alignments. Using Trinity (K-mer =25, group_pairs_distance =500), the clean reads were assembled to produce a collection of contigs representing the pool of cDNA fragments found in this study. The resultant contigs were further joined into scaffolds (transcripts) using the read mate pairs. The overlap settings used in this process were 31 bp and 95% similarity, with all other default parameters. After assembly, clustering was performed on scaffolds to yield a unigene dataset using TGI Clustering tools [79]. The quality of the assembly was benchmarked against the core set of eukaryotic genes using the Core Eukaryotic Genes Mapping Approach (CEGMA) algorithm (*E* value cutoff ≤1e-5) [55].

### Sequence bioinformatics analysis

Open reading frames (ORFs) were predicted using the “getorf” program of the EMBOSS software package [80], with the longest ORF extracted from each unigene. ORF lengths were determined from the first methionine codon to the next stop codon. For incomplete sequences where the ORF abutted the 5′ or 3′ end, the length was determined from the relevant end. Signal peptide and cleavage site predictions were conducted using SignalP (v3.0) [81].

Several complementary approaches were utilized to annotate the sequences. The initial comparisons of *P. cactorum* unigenes were carried out against the *P. infestans* genome (http://www.broadinstitute.org/annotation/genome/phytophthora_infestans/MultiHome.html), *P. sojae* genome (http://genome.jgi-psf.org/Physo1_1/Physo1_1.home.html), *Pythium* genome (http://pythium.plantbiology.msu.edu/), NCBI non-redundant nucleotide sequence (Nt) and non-redundant protein (Nr) (http://www.ncbi.nlm.nih.gov/), Swiss-Prot/TrEMBL (http://www.uniprot.org/), InterProScan (http://www.ebi.ac.uk/interpro/scan.html) databases. Functional annotation by gene ontology terms (GO; http://www.geneontology.org) was analyzed by Blast2GO software [9, 82, 83]. The COG annotation was performed to predict and classify gene functions using Blastall software against Cluster of Orthologous Groups database (http://www.ncbi.nlm.nih.gov/COG/). In order to gain an overview of gene pathways represented in the transcriptome, pathway analysis was performed using the online Kyoto Encyclopedia of Genes and Genomes (KEGG) Automatic Annotation Server (KAAS). The bi-directional best hit (BBH) method was used to obtain KEGG Orthology (KO) assignments [84]. All searches were performed using BLASTx [84] with *E*-value cut-off of 1e-5.

An all-against-all BLASTp (*E*-value cutoff of 1e-10) [85] similarity search was performed amongst predicted protein sequences of four *Phytophthora* species, *P. cactorum, P. infestans*, *P. sojae*, and *P. ramorum* (http://genome.jgi-psf.org/Phyra1_1/Phyra1_1.home.html). The pairwise similarities generated by this analysis were parsed and stored in a matrix that served as input for clustering by the TribeMCL approach [86]. The groups of homologous proteins were clustered using Markov cluster (MCL) algorithm at an inflation rate (I) of 2.0 and other default settings.

RXLR and CRN effectors were initially predicted based on the BLASTx comparisons (*E* value ≤1e-5) against *P. infestans*, *P. sojae* genomes and NCBI databases. Both best hit and effector sequences were manually examined. In a second approach to find such effectors, the RXLR effector identification pipeline and the HMM seach were used as described previously [13, 56] with minor modification. In brief, an RXLR motif was defined within the first 100 residues. Using the collection of CRN effectors of *Phytophthora* species [13], similar searches were also performed to identify these effectors in *P. cactorum* dataset. To search for the conserved WY-domain, HMMER [87] was used to search the C-terminal sequences (downstream of the RXLR motif) of the predicted RXLR effectors using the previously described HMM models [38]. Hits that scored greater than an HMM score of 0.0 were considered putative WY-domain-containing proteins.

### RT-PCR and qRT-PCR analyses

RNA extracted from at least two biological replicates was used for cDNA synthesis employing the M-MLV Reverse Transcriptase (RNase H Minus) and random hexamer primers (TaKaRa) following the manufacturer’s protocol. Primers (Additional file 12) complementary to the genes were designed based on the assembled *P. cactorum* unigene sequences. RT-PCR was carried out on a thermo cycler LS-96G (Thermofisher, Dubuque, IA, USA) with the following programs: 94°C for 5 min, 95°C for 15 s, 46 to 60°C for 30 s and 72°C for 1 min for 29 cycles (40 cycles for qRT-PCR), and, finally melting curve analysis. For qRT-PCR, SYBR Green dye (Applied Biosystem, Foster City, CA, USA) was used according to the instructions. In each case, the internal control for *P. cactorum* transcripts was the ubiquitin-conjugating enzyme-encoding gene *PcUbc* (Unigene ID U13532) and, for qRT-PCR, plant-grown mycelium was used as the calibrator. Fold expression was calculated based on expression in MY and data were analyzed by Student’s *t*-test (*P* < 0.05) for qRT-PCR. To minimize the misinterpretation of results, water controls were employed for all PCR experiments, to verify that no unexpected products were amplified. When *P. cactorum* gene expression was monitored in infected *N. benthamiana* leaves, cDNA from mock-inoculated plants was used as an additional negative control for each primer pair. This was to exclude the possibility that amplification products were derived from plant sequences. All RT-PCRs were performed at least three times.

### Effector gene validation, cloning and multiple sequence alignment

To verify predicted genes, PCR was conducted to test for the presence of an amplified fragment of the predicted size. The PCR reactions were performed using the genomic DNA of *P. cactorum* strain 10300 and specific primers (Additional file 12), with 30 cycles of 30 s at 94°C, 30 s at 55 to 60°C (depending on the gene), and 60 s at 72°C. The PCR products were examined using a 1% agarose gel.

Amplified effector genes were cloned directly into a T-DNA binary vector for transient expression in plant tissue. The gene fragments were amplified from genomic DNA using high-fidelity DNA polymerase (PrimeSTAR® HS DNA Polymerase, TaKaRa) and cloned into the *Sma*I site of the binary potato virus X (PVX) vector pGR107 [88]. Primers (Additional file 12) towards RXLR effectors were designed to amplify sequences encoding the mature RXLR proteins (without signal peptide) together with an in-frame ATG start codon. Primers (Additional file 12) for elicitin and NLP genes were designed to amplify the full-length gene sequences including signal peptide-encoding region. The inserts were verified by sequencing using PVX-F/PVX-R primers [40]. The constructs in pGR107 were introduced independently into *Agrobacterium tumefaciens* strain GV3101 by electroporation. After growing at 28°C on Luria-Bertani (LB) agar plates containing rifampicin (25 μg/ml) and kanamycin (50 μg/ml) for 2 days, individual colonies were extracted with NaOH (2 mM) lysis solution and the inserts amplified to verify that the correct clones had been selected for the agroinfiltration (*A. tumefaciens* infiltration) assay.

Sequence alignment was performed to investigate the relationship of the predicted effectors with other oomycete species using the program BioEdit (v7.2.0) [89]. To search for effector homologues in other oomycete species, in addition to the TribeMCL analysis, BLASTx comparison (*E* ≤1e-5) was also performed against the reference genomes including *Pythium* spp., *P. infestans*, *P. sojae*, *P. parasitica* (http://www.broadinstitute.org/annotation/genome/Phytophthora_parasitica/MultiHome.html) and *P. capsici* (http://genome.jgi-psf.org/Phyca11/Phyca11.home.html), and the protein sequences of other species were downloaded from the respective databases.

### Agroinfiltration assay

*A. tumefaciens* strains containing the pGR107 constructs were grown in LB broth plus appropriate antibiotic at 28°C and 200 rpm for 2 days. The cells were collected by centrifugation (3, 000 × *g*, 5 min), washed three times in 10 mM MgCl_2_, and resuspended in 10 mM MgCl_2_ to an optical density at 600 nm of 0.4 to 0.6, and then incubated at room temperature for 1 to 3 h prior to infiltration. Small nicks were made with a sterile needle on the abaxial side of leaves from *N. benthamiana* plants and then 20 to 40 μl of cell suspension carrying the respective constructs was infiltrated through each nick using a blunt syringe. *A. tumefaciens* strains carrying green fluorescent protein (*GFP*) and the *P. infestans* elicitin *INF1*[63] genes were employed as negative and positive controls, respectively. Each assay consisted of at least five plants inoculated on four leaves (total of twenty leaves). Symptom development was monitored visually 3 to 10 d after infiltration. All of the experiments were repeated at least five times, producing similar results in each case.

### Polymorphism analysis

Two pairs of oligonucleotide primers, PcRXLR6snp for *PcRXLR6* and PcRXLR7snp for *PcRXLR7*, were designed for the amplifications of fragments containing the entire ORFs of the genes (Additional file 12). The fragments were cloned into a TA vector pMD-18 T (TaKaRa). The genomic DNA from each of seven *P. cactorum* isolates (Additional file 14) was used for PCR amplification. Single nucleotide polymorphisms (SNPs) were confirmed as follows. First, SNPs recovered independently from more than one strain were judged to be valid. Second, all SNPs, including the SNPs that were only detected in one strain, were confirmed by analyzing chromatographs obtained by sequencing amplicons from two independent PCR amplifications of genomic DNA.

Protein sequences were aligned using ClustalW [90]. Synonymous and non-synonymous substitution rates (d*S* and d*N*, respectively) were determined using the Yn00 program in PAML (v4.7) [64]. The statistical significance of the difference between d*N* and d*S* was estimated using the standard errors output from PAML for Student’s *t*-test with a *P* value <0.05. To identify which amino acid sites of effectors have been affected by diversifying selection, maximum likelihood models of codon substitution that allow for selection pressures among sites along the protein were used. Analyses were done using the program CODEML in the PAML package [64]. Codons under positive selection for model M8 were identified using a Bayes Empirical Bayes approach (BEB) [91–93] and considering a posterior probability of >95%.

### Availability of supporting data

The Illumina sequencing reads presented in this study have been deposited in the NCBI Sequence Read Archive database under the study number SRP040619. The assembled *P. cactorum* unigene sequences (21,662) were deposited in the NCBI Transcriptome Shotgun Assembly sequence database under the accession GBGX00000000. The version described in this paper is the first version, GBGX01000000. The full-length gene ORF sequences of *PcNLP2*, *PcNLP3, PcNLP1*, *PcELL1*, *PcUbc* and *PcapRXLR7* are available through GenBank accession numbers KM527901, KM527902 and KM068096 - KM068099. Other supporting data are included as additional files.

## Electronic supplementary material

Additional file 1: **mRNA expression of selected RXLR effector candidate genes in** ***P. cactorum*** **is up-regulated during the cyst germination.** The cysts were germinated on a cellophane membrane that was placed on the top of an *N. benthamiana* leaf (A - B, artificial condition), or directly on *N. benthamiana* leaves (C, natural infection). In (A), the gene expression levels were determined by calculating the number of reads for each gene and then normalized to RPKM. RPKM integer values (numbers on the respective bars) were used to build the plot. In (B) and (C), the gene expression levels were displayed as fold mycelium expression. *PcUbc* was used as the internal control for qRT-PCR analysis. Error bars indicate the standard error and the letters “a” and “b” indicate the significance in Student’s *t*-test. This experiment in each case was performed with three biological replicates. MY, vegetative mycelium; GC, germinating cyst. (TIFF 916 KB)

Additional file 2: **Summary of Illumina transcriptome sequencing for** ***P. cactorum*** **.** (DOC 26 KB)

Additional file 3: **Overview of the** ***P. cactorum*** **transcriptome sequencing and assembly.** (A) Length distribution of *P. cactorum* transcripts. (B) Size distribution of *P. cactorum* unigene coding regions. (C) Log plot showing the dependence of unigene lengths on the number of assembled reads. (TIFF 6 MB)

Additional file 4: **Summary of the genomic sequences of** ***P. cactorum*** **deposited in NCBI mapping to** ***P. cactorum*** **unigenes by BLASTn analysis.** (XLS 164 KB)

Additional file 5: **Summary of the ESTs of** ***P. cactorum*** **deposited in NCBI mapping to** ***P. cactorum*** **unigenes by BLASTn analysis.** (XLS 37 KB)

Additional file 6: **Functional annotation of** ***P. cactorum*** **assembled sequences and** ***P. infestans*** **PITG genes using gene ontology (GO) terms.** GO analysis was performed using general (level 2) terms from the three ontologies (cellular component, molecular function and biological process). PITG, *P. infestans* strain T30-4 gene models. Trans, *P. cactorum* transcriptome unigenes. (TIFF 3 MB)

Additional file 7: **Clusters of orthologous groups (COG) classification.** In total, 5,491 of the 18,624 *P. cactorum* unigenes with Nr hits were grouped into 25 COG classifications. (TIFF 2 MB)

Additional file 8: **Complete list of KEGG biochemical pathways for** ***P. cactorum*** **transcriptome.** (XLS 85 KB)

Additional file 9: **The summary of 94** ***P. cactorum*** **RXLR effector candidates.** (XLS 102 KB)

Additional file 10: **BLASTx searches of** ***P. cactorum*** **RXLR effector candidates against** ***P. infestans*** **and** ***P. parasitica*** **genome databases.** (XLS 36 KB)

Additional file 11: **The summary of 64** ***P. cactorum*** **CRN effector candidates.** (XLS 80 KB)

Additional file 12: **List of primers used for PCR validation, (q)RT-PCR, PVX cloning and polymorphism analysis of the** ***P. cactorum*** **genes.** (XLS 34 KB)

Additional file 13: **Multiple sequence alignment of effector protein sequences from** ***P. cactorum*** **against different** ***Phytophthora*** **species and** ***Py. ultimum*** **.** Protein sequences were aligned and shaded for consensus (60% threshold for shading) using BioEdit. (A)-(D) The RXLR effectors PcRXLR6, PcRXLR7, PcRXLR13 and PcRXLR27 in order. Red boxes denoted putative RXLR and EER motifs. (E) The NLP effector PcNLP1. Blue arrows indicated conserved cysteines and key functional residues previously described [19]. The putative GHRHDWE motif was denoted by red box. (F) The elicitin PcELL1. The numbers following the organism names “*P. sojae*” or “*P. capsici*” are the homologue protein IDs from the corresponding species genomes. PITG and PPTG, *P. infestans* and *P. parasitica* gene models, respectively; PYU1, *Py. ultimum* gene models. (TIFF 2 MB)

Additional file 14: ***P. cactorum*** **isolates used in this study.** (DOC 34 KB)
