# Supplementary figures and images for: Transcriptomic analysis of the phytopathogenic oomycete Phytophthora cactorum provides insights into infection-related effectors
Source: BMC Genomics. 2014 Nov 18;15(1):980. doi: 10.1186/1471-2164-15-980 (PMC4289400; doi:10.1186/1471-2164-15-980)

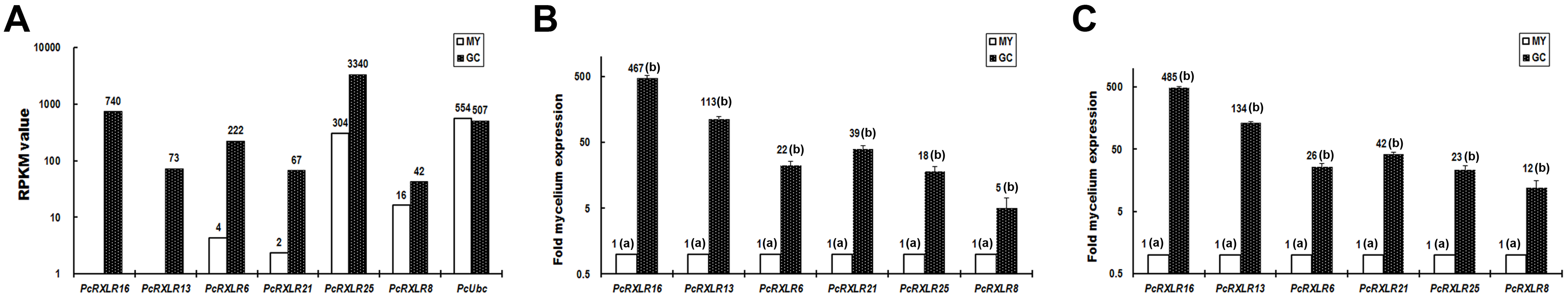

Supplement: Supplementary file 1 — Additional file 1: mRNA expression of selected RXLR effector candidate genes in P. cactorum is up-regulated during the cyst germination. The cysts were germinated on a cellophane membrane that was placed on the top of an N. benthamiana leaf (A - B, artificial condition), or directly on N. benthamiana leaves (C, natural infection). In (A), the gene expression levels were determined by calculating the number of reads for each gene and then normalized to RPKM. RPKM integer values (numbers on the respective bars) were used to build the plot. In (B) and (C), the gene expression levels were displayed as fold mycelium expression. PcUbc was used as the internal control for qRT-PCR analysis. Error bars indicate the standard error and the letters “a” and “b” indicate the significance in Student’s t-test. This experiment in each case was performed with three biological replicates. MY, vegetative mycelium; GC, germinating cyst. (TIFF 916 KB) [file 12864_2014_6857_MOESM1_ESM.tiff]

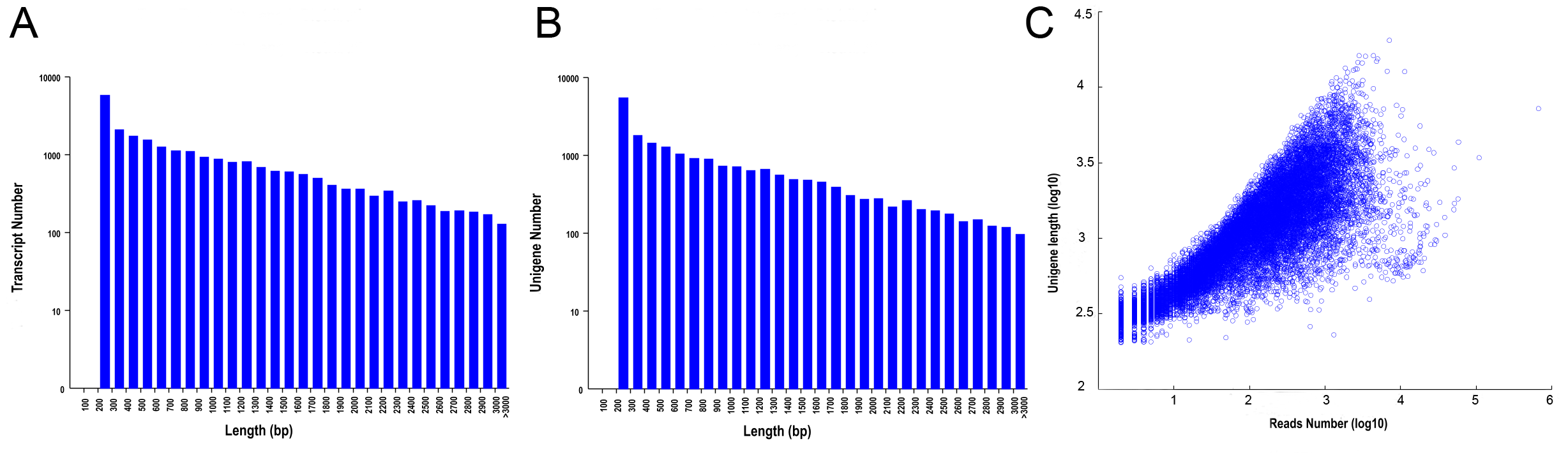

Supplement: Supplementary file 3 — Additional file 3: Overview of the P. cactorum transcriptome sequencing and assembly. (A) Length distribution of P. cactorum transcripts. (B) Size distribution of P. cactorum unigene coding regions. (C) Log plot showing the dependence of unigene lengths on the number of assembled reads. (TIFF 6 MB) [file 12864_2014_6857_MOESM3_ESM.tiff]

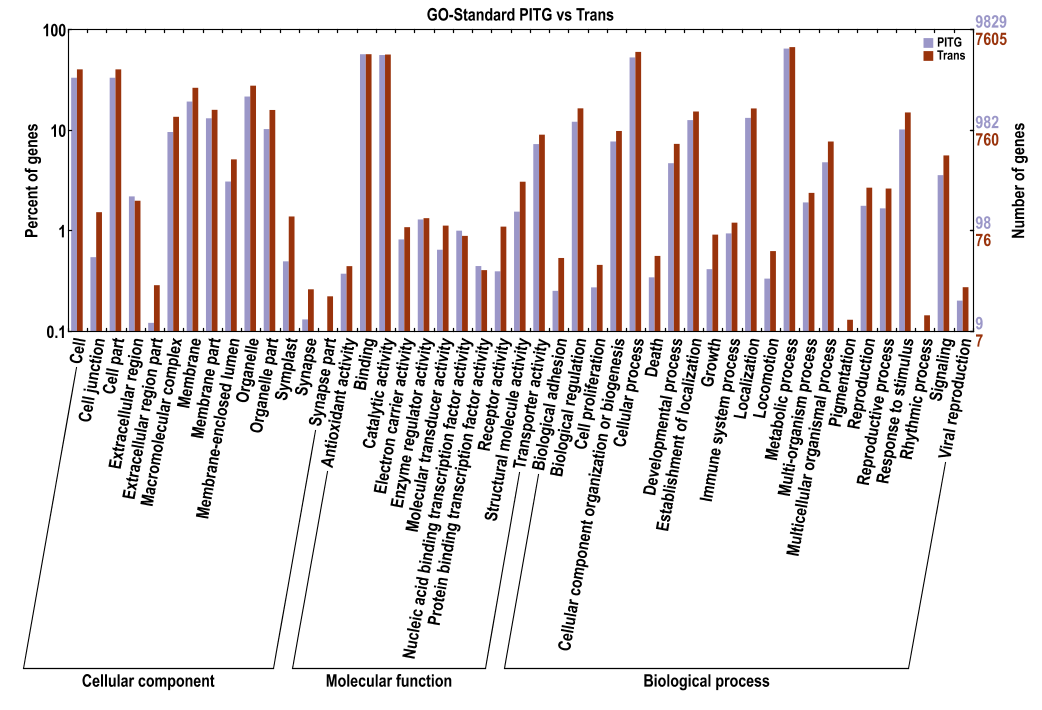

Supplement: Supplementary file 6 — Additional file 6: Functional annotation of P. cactorum assembled sequences and P. infestans PITG genes using gene ontology (GO) terms. GO analysis was performed using general (level 2) terms from the three ontologies (cellular component, molecular function and biological process). PITG, P. infestans strain T30-4 gene models. Trans, P. cactorum transcriptome unigenes. (TIFF 3 MB) [file 12864_2014_6857_MOESM6_ESM.tiff]

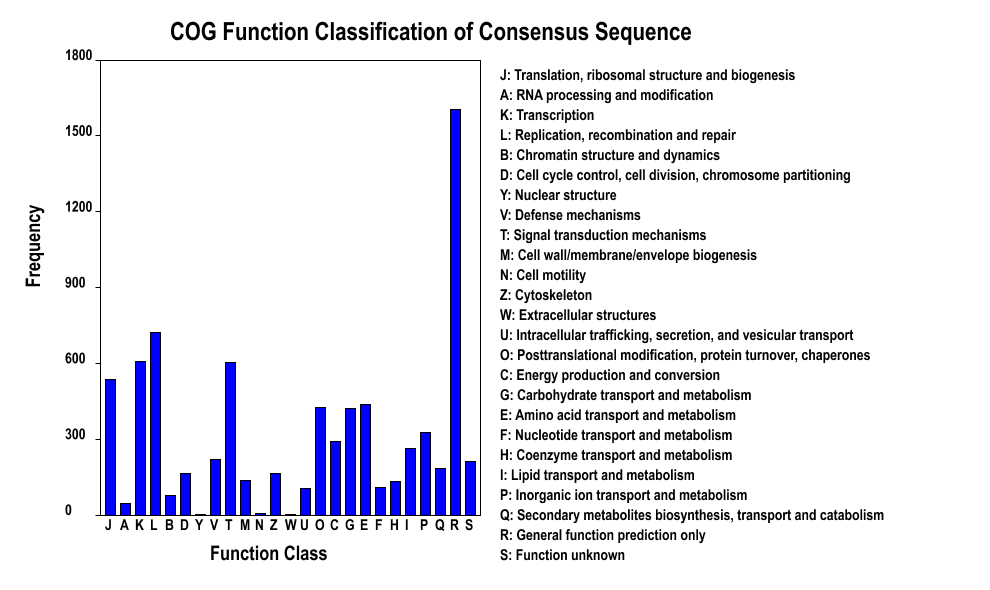

Supplement: Supplementary file 7 — Additional file 7: Clusters of orthologous groups (COG) classification. In total, 5,491 of the 18,624 P. cactorum unigenes with Nr hits were grouped into 25 COG classifications. (TIFF 2 MB) [file 12864_2014_6857_MOESM7_ESM.tiff]

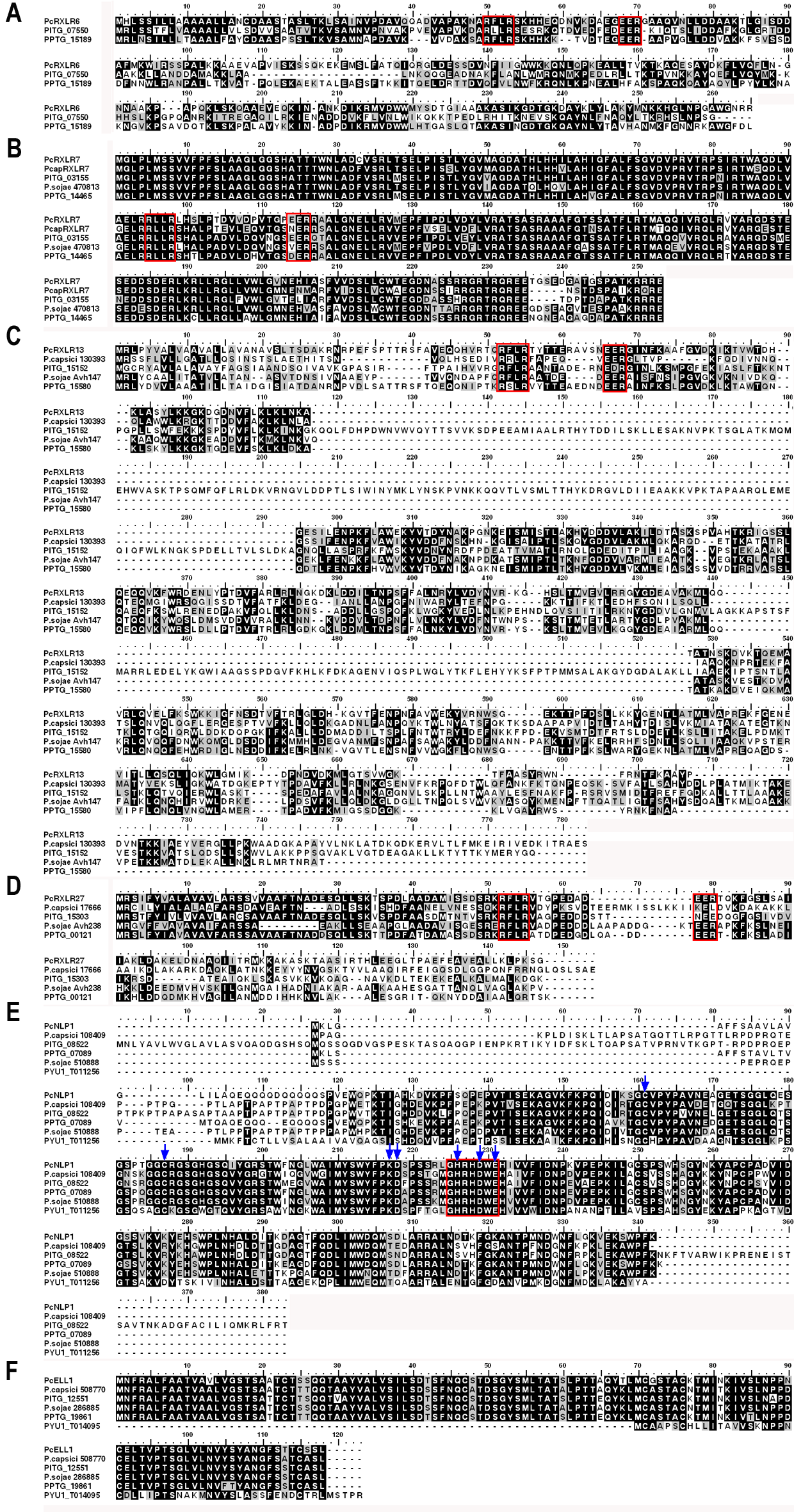

Supplement: Supplementary file 13 — Additional file 13: Multiple sequence alignment of effector protein sequences from P. cactorum against different Phytophthora species and Py. ultimum . Protein sequences were aligned and shaded for consensus (60% threshold for shading) using BioEdit. (A)-(D) The RXLR effectors PcRXLR6, PcRXLR7, PcRXLR13 and PcRXLR27 in order. Red boxes denoted putative RXLR and EER motifs. (E) The NLP effector PcNLP1. Blue arrows indicated conserved cysteines and key functional residues previously described [19]. The putative GHRHDWE motif was denoted by red box. (F) The elicitin PcELL1. The numbers following the organism names “P. sojae” or “P. capsici” are the homologue protein IDs from the corresponding species genomes. PITG and PPTG, P. infestans and P. parasitica gene models, respectively; PYU1, Py. ultimum gene models. (TIFF 2 MB) [file 12864_2014_6857_MOESM13_ESM.tiff]
