# Supplementary material for: Transcriptomic analysis of the phytopathogenic oomycete Phytophthora cactorum provides insights into infection-related effectors
Source: BMC Genomics. 2014 Nov 18;15(1):980. doi: 10.1186/1471-2164-15-980 (PMC4289400; doi:10.1186/1471-2164-15-980)
Supplement: Supplementary file 2 — Additional file 2: Summary of Illumina transcriptome sequencing for P. cactorum . (DOC 26 KB) [file 12864_2014_6857_MOESM2_ESM.doc]

**Additional file 2 Summary of Illumina transcriptome sequencing for *P. cactorum***

| **Library** | **Cycle number** | **Total reads** | **Total bases** | **GC content (%)** | **Cycle Q20 (% )** |
| --- | --- | --- | --- | --- | --- |
| 1 | 75 | 12,969,224 | 972,691,800 | 56.31 | 94.46 |
| 2 | 83 | 15,724,668 | 1305,147,444 | 57.07 | 95.49 |
