# Supplementary material for: Transcriptomic analysis of the phytopathogenic oomycete Phytophthora cactorum provides insights into infection-related effectors
Source: BMC Genomics. 2014 Nov 18;15(1):980. doi: 10.1186/1471-2164-15-980 (PMC4289400; doi:10.1186/1471-2164-15-980)
Supplement: Supplementary file 14 — Additional file 14: P. cactorum isolates used in this study. (DOC 34 KB) [file 12864_2014_6857_MOESM14_ESM.doc]

**Additional file 14 *P. cactorum* isolates used in this study**

| **Isolate ID** | **Host** | **Origin** | **Contributor** |
| --- | --- | --- | --- |
| 10300 ^a^ | Strawberry | Ås, Norway | May Bente Brurberg, Bioforsk, Norway |
| 19447 | Strawberry | Ås, Norway | May Bente Brurberg, Bioforsk, Norway |
| Fin1 | Strawberry | Finland | May Bente Brurberg, Bioforsk, Norway |
| PC7 | Pear | Nanjing, China | Yuanchao Wang, NJAU, China |
| PH-1-14-04 | Apple | Fars Province, Iran | Zia Banihashemi, Shiraz University, Iran |
| PH-1-8-03 | Walnut | Fars Province, Iran | Zia Banihashemi, Shiraz University, Iran |
| PH-1-17-05 | Walnut | Fars Province, Iran | Zia Banihashemi, Shiraz University, Iran |

^a^ Reference strain used for transcriptome sequencing
